# Mediating role of food web structure in linking diversity to multidimensional stability: Evidence from global marine ecosystems

**DOI:** 10.1126/sciadv.adv3841

**Published:** 2025-10-10

**Authors:** Jianfeng Feng, Xuhao Wan, Ruyue Wang, Shengpeng Li, Maohong Wei, Vasilis Dakos, Marcos Llope, Xueqiang Lu, Nils Chr. Stenseth

**Affiliations:** ^1^ College of Environmental Science and Engineering, Nankai University, Tianjin, P. R. China.; ^2^ Tianjin Foreign Studies University, Tianjin, P. R. China.; ^3^ Institut des Sciences de l’Evolution de Montpellier (ISEM), CNRS, University of Montpellier, Montpellier, France.; ^4^ Instituto Español de Oceanografía (IEO-CSIC), Centro Oceanográfico de Cádiz, Puerto Pesquero, Muelle de Levante, E-11006, Cadiz (Andalusia), Spain.; ^5^ Centre for Ecological and Evolutionary Synthesis (CEES), Department of Biosciences, University of Oslo, NO-0316 Oslo, Norway.

## Abstract

The diversity-stability relationship persists as a central ecological debate, as existing work primarily examines direct diversity and structural drivers, while empirical evidence clarifying how direct and structure-mediated pathways jointly shape ecosystem multistability remains limited. Here, we analyzed 217 global marine food webs, quantifying structural properties and multidimensional stability to evaluate how diversity and structure jointly influence stability. Our analyses reveal that diversity is consistently linked to stability through dual pathways: positively associated with resistance and resilience via indirect structural mediation, yet negatively correlated with local stability unless interaction strength is accounted for. Critically, omitting structural mediation yields a net negative diversity-stability correlation, whereas integrating food web structure uncovers context-dependent positive relationships, underscoring structural metrics as pivotal explanatory variables. Our findings reconcile the diversity-stability debate by showing that the food web structure mediates context-dependent stability outcomes—integrating direct and indirect pathways resolves contradictions and advances actionable metrics for conservation strategies resilient to environmental changes.

## Teaser

The marine food web structure mediates diversity-stability relationships.

## INTRODUCTION

Ecosystem stability has long fascinated ecologists, particularly its relationship with species diversity (*1*, *2*). Before 1970, it was widely believed that greater diversity inherently bolstered stability (*3*, *4*). May’s analysis overturned this view: Using random community matrices, it showed that increases in species richness, connectivity, and interaction complexity can undermine local (asymptotic) stability (*5*). Critics soon pointed out that the stochastic community matrices were inadequate for demonstrating the stability-complexity relationship of ecosystems, as they omitted primary-producer diversity and realistic trophic architecture (*6*, *7*), promoting the development of empirically grounded food web models (*8*). In food web research, taxa are often aggregated into “trophic species” or living groups—organisms sharing identical predator-prey links (*9*, *10*). Although coarser than species and finer than trophic levels, this approach minimizes biases from uneven taxonomic resolution (*9*). Empirical studies report both positive and negative correlations between living-group richness and stability (*11*, *12*). These models reaffirmed the practical significance of the diversity-stability relationship (*13*).

In addition to diversity, other food web structure descriptors are regarded as also crucial factors with regard to stability. While some studies have tested the hypothesis of a negative correlation between connectivity and stability (*11*, *12*, *14*, *15*), with conflicting results, others have found that connectivity enhances stability (*10*, *16*). In contrast to May’s study, Haydon (*17*) demonstrated that increased interaction strength enhances stability, when less regulated ecosystem elements are combined with more tightly regulated elements. Subsequent studies have also demonstrated that the relationship between interaction strength and stability is contingent upon the size of the food web (*12*). Furthermore, other food web structure indicators have been demonstrated to influence ecosystem stability. For example, omnivory has a stabilizing effect on food webs (*18*) and enhances resilience (*19*). In addition, high-cycling systems exhibit low resilience due to constrained nutrient inputs (*20*). Omnivory often enhances stability and recovery (*18*, *19*). However, there are fewer studies that combine different indicators.

Most of the current research focuses on elucidating the direct relationship between diversity and stability, as well as the relationship between food web structure and stability. One of the fundamental questions that ecologists have sought to address is how interactions within food webs vary in accordance with the number of species involved (*21*). May (*5*) posited that diverse ecosystems result in fewer or weaker species interactions. This is contradicted by findings that the number of interactions increases linearly with the number of species (*22*). These findings imply that diversity may drive stability directly or indirectly by mediating food web structure. The absence of empirical evidence has resulted in a paucity of research examining the relationships between these patterns (*23*). It is therefore crucial to conduct a joint investigation into the effects of diversity and food web structure on the stability metrics of empirical ecosystems in response to perturbations.

Bridging this theoretical-empirical divide demands a multidimensional approach to stability (*24*). Theoretical work typically emphasizes asymptotic, or local stability (*5*, *11*, *25*), quantified by the dominant eigenvalue of the interaction matrix and indicating the rate at which a system returns to equilibrium following small perturbations (*26*). Empirical research, by contrast, measures “resistance”—the degree to which ecosystem structure and function endure during disturbance—and “resilience”—the speed and extent of recovery once equilibrium has shifted (*27*, *28*). Reliance on a single metric or the reduction of these dimensions to a univariate index can yield conflicting diversity-stability relationships and obscure the mechanisms that underpin stability (*24*). Thus, the adoption of explicit, multidimensional stability metrics is essential for reconciling divergent findings and advancing our understanding of ecosystem dynamics.

Most research treats stability as unidimensional—focusing on local stability, resistance, or resilience—obscuring how diversity influences stability directly versus indirectly via network structure (*23*). To disentangle these pathways, we analyzed 217 global marine food webs constructed under the standardized Ecopath framework (*29*). Ecopath models integrate empirical data on biomass, production, consumption, and diet composition into interaction matrices, enabling realistic stability assessments (table S1) (*13*). We computed three stability metrics: (i) local stability, the negative real part of the largest characteristic root of the community interaction matrix in the Ecopath model; (ii) resistance, the maximum percentage change in biomass under stochastic mortality disturbance; and (iii) resilience, the percentage biomass recovery 1 year after disturbance cessation via Ecosim simulations. These methods have been used in other studies and are comparable (*30*, *31*). Last, we applied generalized linear mixed-effects models and piecewise structural equation modeling (SEM) to evaluate how diversity and food web structure jointly govern multidimensional stability.

## RESULTS

### Global patterns of marine food web stability metrics, diversity, and typical food web structure indicators

**Fig. 1.**
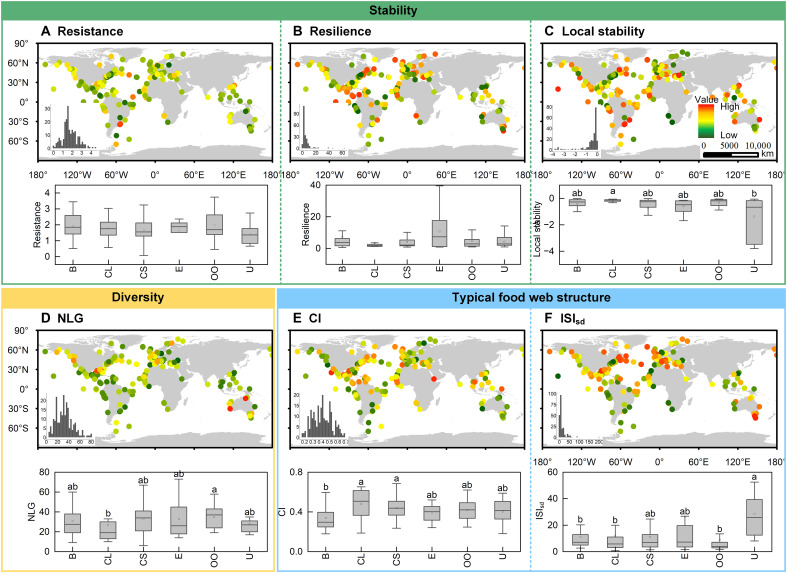
Global distribution of marine food web stability metrics, diversity, and typical food web structure indicators. Stability indicators included resistance (**A**), resilience (**B**), and local stability (**C**). Diversity refers to the NLGs (**D**). Typical food web structure indicators included connectance index (CI) (**E**) and standard deviation of interaction strength index (ISI_sd_) (**F**). The inset image in the lower left corner of each map represents a histogram of the distribution of the indicator ranges. Different lowercase letters in the test of variance plots indicate statistically significant differences at the 0.05 level. The box plot shows the quartiles of the indicator. The horizontal coordinates of the box plot refer to ecosystem types. B, bay; CL, coastal lagoon; CS, continental shelf; E, estuary; OO, open ocean; U, upwelling ecosystems.

The calculation of three stability metrics for each of the 217 marine ecosystems permitted the illustration of their global distribution, as shown in Fig. 1. The resistance of marine food web models exhibited a Gaussian distribution, with most values concentrated around 2 (Fig. 1A). Conversely, the distribution of resilience was markedly skewed (Fig. 1B), with most values below 10. Furthermore, the local stability of the majority of food webs demonstrated a left-skewed distribution (Fig. 1C). No statistically significant differences were observed in resistance and resilience among different ecosystems, indicating that their global distribution was across ecosystems. However, local stability was found to be statistical significantly lower in upwelling ecosystems compared to coastal lagoons, with considerable variation between ecosystems (Fig. 1, A to C).

The marine food webs under investigation are composed of 6 to 81 NLGs (number of living groups) (Fig. 1D), with connectance indices (CIs) spanning from 0.1 to 0.7 (Fig. 1E). The standard deviation of interaction strength index (ISI_sd_) was skewed, with a predominant concentration below 25 (Fig. 1F). The NLGs in coastal lagoons exhibited a statistically significantly lower concentration than those in the open ocean. The CI of bays demonstrated a statistically significantly lower concentration than that of coastal lagoons and continental shelves, while the ISI_sd_ of upwellings exhibited a statistically significantly higher concentration than that of bays, coastal lagoons, and the open ocean.

### Relationships among diversity, food web structure, and stability metrics

**Fig. 2.**
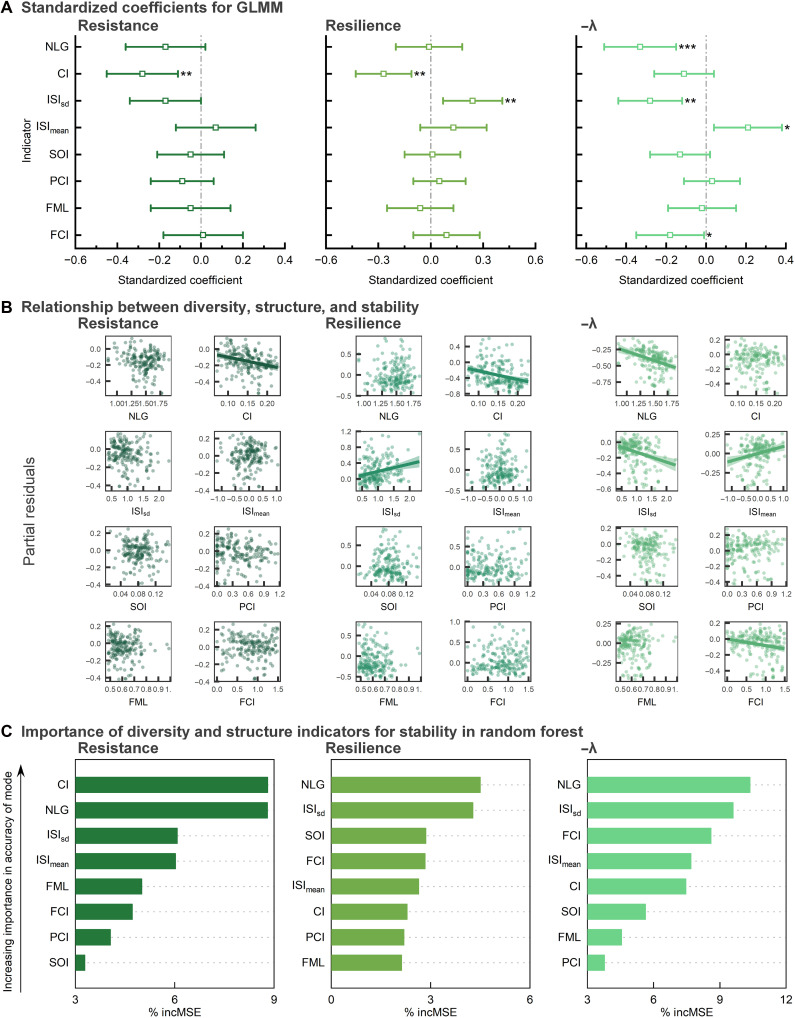
Relationship among diversity, food web structure, and stability. (**A**) Standardized coefficients for generalized linear mixed-effects models. (**B**) Scatterplots and fits between diversity, structure indicators, and partial residuals of stability. (**C**) Order of importance of variables in the random forest model. *P < 0.05, **P < 0.01, and ***P < 0.001. The error bars show a 95% confidence interval. Scatterplots refer to the fit of the bias parameter of the stability metrics to all metrics. Indicators with statistically significant effects were fitted. All horizontal coordinates were log-transformed, and vertical coordinates were log-transformed partial residuals. The shaded regions show the 95% confidence interval. The %incMSE (increase in mean squared error) indicates the error in model predictions after the predictor variables were randomly substituted. ISI_mean_, mean of interaction strength index; SOI, system omnivory index; PCI, predator cycle index; FML, Finn’s mean path length.

A negative correlation was observed between resistance and CI (Fig. 2, A and B). A negative correlation was also observed between resilience and CI, while a positive correlation was evident between resilience and ISI_sd_ (Fig. 2, A and B). The correlation between local stability and the remaining variables was as follows: negative correlations with NLG, ISI_sd_, and Finn’s cycling index (FCI); positive correlation with mean of interaction strength index (ISI_mean_; Fig. 2, A and B). No statistically significant correlations were identified between the remaining indicators and the various aspects of stability under investigation. The partial correlation between stability (resistance and resilience) at different quantiles and diversity and structure indicators also indicated that it was reasonable to use the median of the stability of all living groups to characterize the overall stability of the ecosystem (fig. S1). The results of the random forest model indicate that NLG plays a notable role in all three types of stability, with ISI_sd_ also demonstrating a notable relationship on resilience and local stability. Furthermore, the results demonstrated that CI had the most notable relative on resistance (Fig. 2C).

### Mediating role of food web structure in diversity-stability relationships

**Fig. 3.**
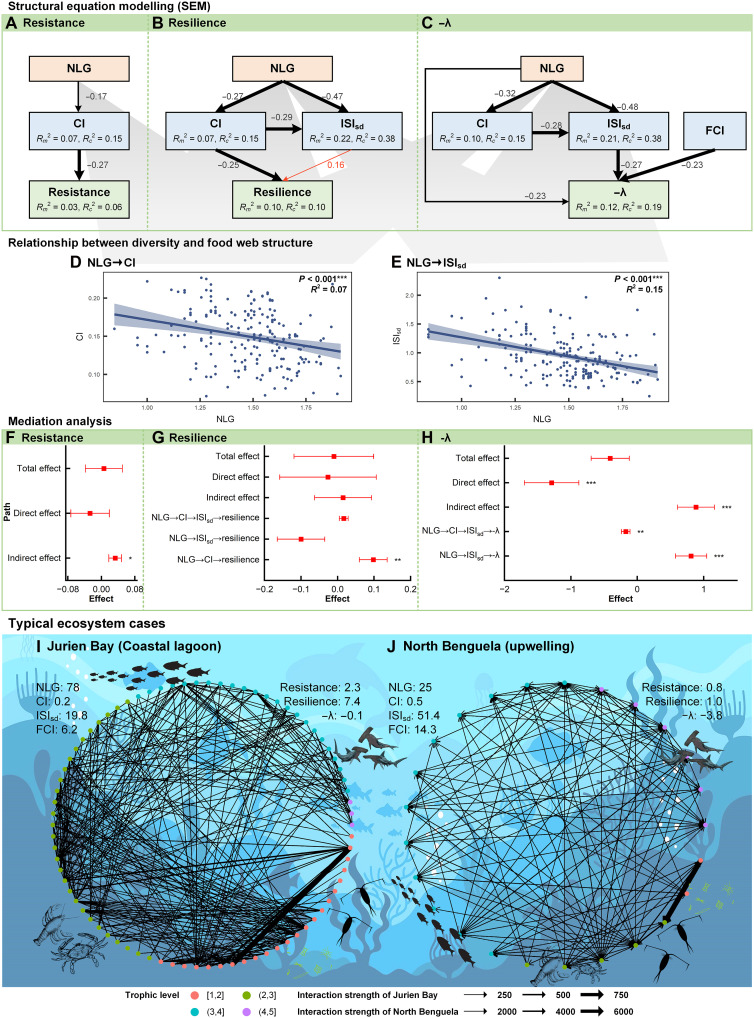
Mediating role of food web structure in diversity-stability relationship. (**A** to **C**) Structural equation models with different stability metrics; only statistically significant paths are shown. Black and red arrows indicate negative and positive causality, respectively. Values associated with arrows indicate standardized path coefficients. The size of the path indicates the path’s significance, with thin to thick indicating significance at the 0.05, 0.01, and 0.001 levels, respectively. *R*^2^*_m_* denotes the proportion of each dependent variable that is explained by the fixed effects. *R*^2^*_c_* denotes the proportion of each dependent variable that is explained by both fixed and random effects. (**D** and **E**) Relationship between diversity and food web structure. (**F** to **H**) Mediation analyses. *P < 0.05, **P < 0.001, and ***P < 0.001. All the data have been log-transformed. (**I** and **J**) Schematic representation of food web structure in two differentiated typical ecosystems (coastal lagoon versus upwelling).

As demonstrated in Fig. 3, the direct and indirect relationship exists between diversity and stability. It is evident that all stability indicators exhibit an indirect relationship with diversity, rather than a direct relationship. In the SEM, resistance has been demonstrated to have a direct negative correlation with CI, while CI is negatively correlated with NLG (Fig. 3A). NLG has been found to be indirectly correlated with resilience, with CI and ISI playing different roles in this process. CI has been demonstrated to have a negative correlation with resilience, while ISI_sd_ has a weaker positive correlation (Fig. 3B). In contrast, NLG has been demonstrated to be associated with local stability directly, as well as indirectly through its influence on food web structure (Fig. 3C). Furthermore, NLG has been observed to be particularly prevalent in more sparse food webs (Fig. 3, D and E). Consequently, when CI was used as a mediating variable, NLG exerted a significant indirect influence on resistance and resilience (Fig. 3, F and G). Despite the existence of a direct and significant negative correlation between local stability and NLG, the indirect associations must not be disregarded, particularly the positive correlation of NLG with local stability through ISI_sd_ (Fig. 3H). These results were largely consistent across nine hypothetical scenarios of varying intensity and duration of random disturbances (figs. S2 and S3).

In conclusion, the larger the number of living groups of the food web, the more resistant and resilient it is to actual large disturbances. Correspondingly, the effect of food web size on local stability may depend on the relative contribution of indirect and direct influences. To better illustrate the relationship between diversity, food web structure characteristics, and stability, a schematic diagram of the food web was constructed for the two typical ecosystems under consideration (Fig. 3, A and B). It is notable that Jurien Bay exhibited a higher level of biodiversity than North Benguela. Despite this, the food web structure of Jurien Bay, as indicated by CI, ISI_sd_, and FCI, was comparatively smaller, suggesting a sparser network. Consequently, Jurien Bay may exhibit greater local stability, as well as enhanced resistance and resilience in the face of huge disturbances.

## DISCUSSION

Our findings demonstrate that marine ecosystems exhibit enhanced stability alongside sparser food web architectures. Whereas previous studies examined the relationship between diversity, connectance, or interaction-strength (ISI_sd_) and stability in isolation, our analysis of 217 marine food webs reveals how these metrics jointly govern stability. NLG correlates negatively with both CI and ISI_sd_. The inverse relationship between diversity and connectance has been identified in numerous studies (*11*, *12*, *32*). Although the diversity’s net effect on resistance and resilience appears neutral, controlling for CI shows that more diverse—and thus sparser connectance—systems better withstand disturbances and recover more rapidly. This negative connectance-stability relationship, first predicted by May and later elaborated by Busiello *et al.* (*5*, *33*), suggests that ecosystems persist either as small, tightly interacting communities or as larger assemblages of weakly linked species. In marine food webs, low connectance optimizes resource flow, enhancing productivity and buffering against perturbations (*32*, *34*). While higher diversity may foster specialized, weaker interactions that diminish local stability, these structural mediations ultimately strengthen multidimensional stability (*5*, *12*, *32*), reconciling discrepancies from single-variable, regression, and path analysis approaches. We must clarify that transient diversity gains may not yield stability benefits unless accompanied by structural reorganization—a process requiring decades in natural systems. Thus, our results highlight structural mediation as a critical pathway for stability but caution against extrapolating short-term diversity changes to rapid stability gains.

The differences between the indicators of stability and structure of various ecosystems were examined. Nevertheless, local stability under small disturbances was highest in coastal lagoon and lowest in upwelling zones. Taking the actual ecosystem of coastal lagoons and upwelling as the examples, as shallow, semi-enclosed systems, coastal lagoons are highly vulnerable to anthropogenic pressures (*35*). The loss of biodiversity in coastal lagoon ecosystems can be attributed to a number of factors, including habitat degradation, coastal urban development, hydrological changes, and pollution (*36*). In contrast, nutrient-rich upwelling regions drive intense bottom-up cascades that amplify species interactions and energy flux, intensifying population oscillations (*37*, *38*). These two indicators represent the primary pathways affecting local stability in SEM. In addition, it was found that there were no statistically significant differences between ecosystems in terms of resistance and resilience, but they were all differently related to diversity and food web structure. Thus, it is clear that no single indicator could fully capture ecosystem stability (*39*, *40*). Resistance and resilience are both essential—particularly in the face of major disturbances (*41*)—yet operate independently: Resilience reflects environmental constraints (*42*), whereas resistance depends on the ecosystem’s initial state (*43*). Thus, a multidimensional perspective is crucial to avoid oversimplification.

The findings of our research have contributed to the ongoing debate on the relationship between diversity, food web structure, and the stability of real ecosystems. In natural ecosystems, food webs are characterized by a paucity of strong interactions, with a prevalence of weaker connections (*44*). This is confirmed by the distribution in Fig. 1. In consideration of nonlinear saturation depletion, nonequilibrium dynamics, and empirical strengths and patterns of interactions, it can be posited that weak interactions serve to enhance stability, due to their capacity to suppress oscillations in the dynamics of predator and prey populations (*18*). The uniform distribution of fluxes over the connecting paths promotes stability (*45*). However, some researchers have also found that although omnivores contribute to the stabilization of food webs, the prevalence of weak interactions is a source of instability (*46*). Conversely, a noteworthy discovery is the inverse relationship between FCI and local stability. FCI is a quantitative measure of the cycle of matter and energy and has been widely applied in ecological research (*47*, *48*). Both local stability and FCI are generated for steady-state networks. The findings of our research suggest that an increase in the cycling of matter and energy in food webs may be associated with a corresponding increase in system instability. In previous studies, ecosystems that had been subjected to disturbance and exhibited instability were observed to have higher FCI values (*49*). Ulanowicz (*50*) believes that an increase in the number of cycles, by cycling between low trophic levels to prevent resource loss as much as possible, represents a steady-state reflection of ecosystem stress. Consequently, the results of present study could validate and support this theory.

While Jacquet *et al.* (*13*) pioneered the analysis of classic complexity descriptors (species richness, connectance, and interaction strength) uncorrelated with stability in empirical food webs, our study extends this field in three key ways: (i) We introduce resistance and resilience, explicitly quantifying the mediating role of food web structure in linking diversity to this multidimensional stability; Jacquet *et al.* (*13*) primarily examined direct relationships with local stability. (ii) By quantifying these stability indicators and additional structural metrics, we reveal their spatial patterns and linear/nonlinear relationships—dimensions unexplored previously. (iii) Integrating 217 modeled marine food webs, we establish that food web structure exerts a moderate, yet substantial, effect on the diversity-multidimensional stability relationship, extending beyond their theoretical framework. This synthesis advances mechanistic understanding of the diversity-stability debate.

Furthermore, we clarify that our proposed metric, the NLGs, is not traditional species richness. Instead, NLG counts functionally aggregated groups sharing identical predator-prey interactions within the model. This represents a deliberate departure from species-centric paradigms. We argue that species-level resolution may not be optimal for analyzing ecosystem stability. Unlike simple species counts (ignoring functional heterogeneity) or broad energy-level classifications (masking horizontal diversity), NLG offers a pragmatic intermediate scale. It balances biological interpretability and analytical tractability: Aggregating trophically identical entities avoids the sensitivity of species counts to functional redundancy while overcoming the oversimplification of coarse nutritional groupings for capturing dynamics. Although NLG does not map directly to specific functional traits, its group-level focus appears more effective at capturing emergent system dynamics than either extreme (individual species or macronutrient groups). While this grouping may mask intragroup ecological differentiation, it provides a unique lens for investigating trophic redundancy and system stability. Sensitivity analysis confirmed that, controlling for resolution, the specific rules defining “surviving” groups do not alter the robustness of the diversity-stability relationship (fig. S6).

Our analysis resolves longstanding contradictions in the diversity-stability debate by demonstrating that food web structure mediates context-dependent stability outcomes. Specifically, biodiversity exhibits dual pathways: It enhances resistance and resilience indirectly by fostering sparser connectance—thereby buffering perturbations—but correlates directly with reduced local stability unless balanced by heterogeneous interaction strengths. Critically, omitting structural mediation masks these compensatory mechanisms, yielding an apparent paradox where diversity destabilizes ecosystems. By integrating multidimensional stability metrics (local stability, resistance, and resilience) with empirically derived network properties, we show that diverse marine food webs achieve stability via structural reorganization—reducing interaction redundancy while maintaining energetically efficient, weak link–dominated topologies. These findings reconcile discrepancies in prior studies by emphasizing that stability emerges from the interplay of diversity, interaction heterogeneity, and connectance scaling. This structural mediation paradigm may offer a roadmap for designing marine protected areas that stabilize ecosystems through network-informed management, rather than static biodiversity targets.

## MATERIALS AND METHODS

### A methodological framework for this study

**Fig. 4.**
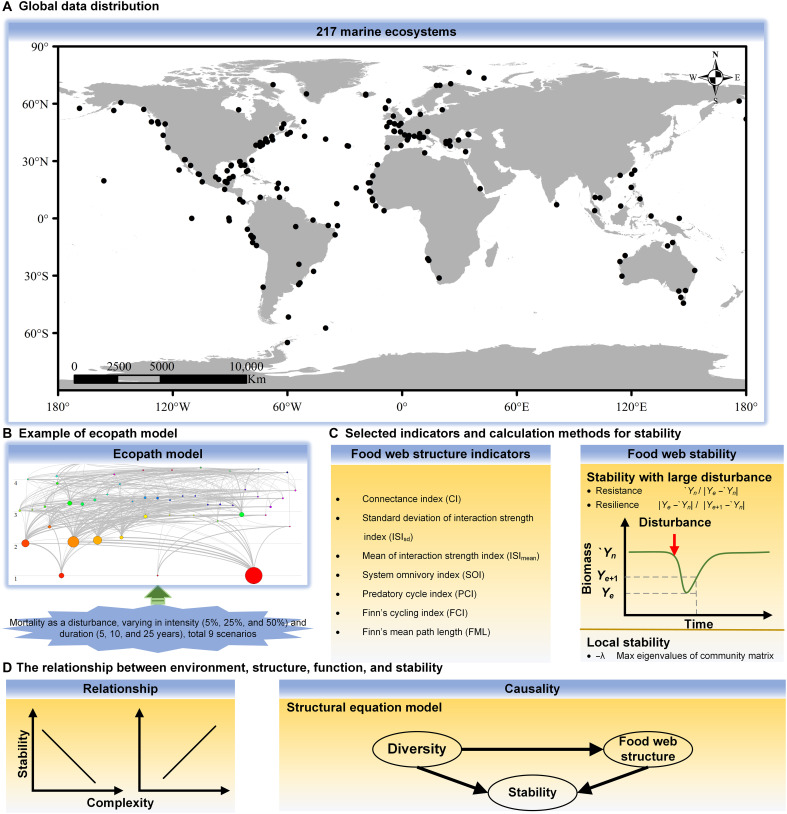
A methodological framework for studying the driving factors of global marine ecosystem stability. (**A**) Global distribution of 217 marine ecosystems. (**B**) Food web structure as estimated by Ecopath. Nine scenarios of Ecosim model simulations with an increase in mortality rate as a disturbance of 5, 25, and 50% intensity, as well as a duration of 5, 10, and 25 years. (**C**) Different structure metrics calculated and screened. On the basis of the food web structure, the local stability was calculated. Two types of stability indicators (resistance and resilience) were calculated on the basis of the results of Ecosim simulations under stochastic disturbances. (**D**) Tests of linear relationships with hypothesized causal paths for piecewise structural equation model.

A total of 217 modeled marine food web datasets from around the world, accessible via EcoBase, were subjected to analysis with a view to determining their local stability and resilience to large-scale disturbances (see Fig. 4A). This is the largest dataset to date of all available marine food web datasets contained in the EcoBase (https://ecobase.ecopath.org/). The application of a consistent methodology and the satisfaction of the Ecopath model framework are characteristics that these marine food web datasets share. The stability of the food webs was calculated by running the Ecopath and Ecosim models with mortality as the disturbance factor (Fig. 4B). Diversity and structure indicators of the food webs were calculated to elucidate the driving mechanisms of stability (Fig. 4C), using a general linear mixed-effects model and a piecewise structure equation model (Fig. 4D).

### EwE food web models

Ecopath with Ecosim (EwE) (*29*), including Ecopath, Ecosim, and Ecospace, is an ecological modeling software based on food web theory. EwE has been widely applied in the assessment and management of aquatic ecosystems throughout the world recently (*51*). In the past years, it has also been incorporated into more traditional stock assessment schemes (*52*).

Ecopath is a static mass balance model, with emphasis on describing the structure and energy flows of aquatic food webs and evaluating ecosystem characteristics. The model assumes that an ecosystem is composed of a series of functional groups, all of which represent the major trophic groups and cover the entire process of energy flow in the ecosystem. The model assumes that the energy input and output of each functional group are balanced, so that the model principle is as follows

$${\left(\frac{P}{B}\right)}_{i}\times B_{i}\times {\text{EE}}_{i}-\sum_{j=1}^{n}B_{j}\times {\left(\frac{Q}{B}\right)}_{i}\times {\text{DC}}_{\mathrm{ij}}-Y_{i}-E_{i}-{\text{BA}}_{i}=0$$

where biomass (*B*) comes from direct estimates such as trawls, visual censuses, plankton sampling, etc. *P/B* (production/biomass ratio) can use estimates of total mortality as input, and for most data on exploited stocks, this comes from literature studies. For unexploited stocks, this can be analyzed from catch curves using appropriate software. Estimates of *Q/B* (consumption/biomass ratio) can be obtained from studies of stomach contents dynamics in the laboratory, in nature, or by combining laboratory and field data. Independent estimation of EE (ecotrophic efficiency) is difficult, and it is usually necessary to estimate it because of the lack of biomass. If EE must be used as an input, the estimated biomass needs to be compared with estimates from similar ecosystems to obtain a suitable value. That is, the EE needs to be possible (<1) and physiologically realistic (e.g., the EE of phytoplankton may be 0.5). DC*_ij_* (proportion of prey *i* in diet of predator *j*) and *Y* (catches by fleet), along with one of the other six parameters in the table, must always be used as inputs to Ecopath. When running the Ecopath parameterization, if all four basic parameters (*B*, *P/B*, *Q/B*, and EE) are entered, the program will ask if biomass accumulation (BA) is estimated. When no is answered, it will further ask if net migration (*E*) is estimated. DC*_ij_* is often derived from literature studies or laboratory analyses of stomach contents. *Y* comes from catch data (table S2).

Ecosim is a dynamic simulation model, with initial parameters inherited from Ecopath. It incorporates time series data to reconstruct the temporal dynamics of historical population biomasses and predicts future trends in the context of fishing pressure and environmental changes, being able to assess the impact of single or multiple pressures on the ecosystem. The model principle is expressed as

$$\frac{dB_{i}}{\mathrm{dt}}=g\sum_{j=1}^{n}Q_{\mathrm{ij}}-\sum_{j=1}^{n}Q_{\mathrm{ij}}+I_{i}-\left(M_{i}+F_{i}+e_{i}\right)\times B_{i}$$

where *dB*/*dt* is the biomass growth rate during time interval *t*; *g* represents the net growth efficiency, which is the ratio of *P*/*B* to *Q*/*B*; *I* is the immigration rate; *M* is the natural mortality rate; *F* is the fishing mortality rate; *e* is the emigration rate; *Q* is the consumption rate, with *Q_ij_* representing the predation on prey *i* by predator *j* and *Q_ij_* representing the consumption of prey *i* by predator *j*.

Two hundred seventeen published Ecopath models of marine food webs all over the world were obtained from EcoBase (http://ecobase.ecopath.org/) (*53*, *54*). These models cover coastal lagoons, continental shelves, coral reefs, open oceans, estuaries, bays, and other ecosystem types (*53*, *55*). The biomass of each species in each food web satisfies the above formula. All food web models have undergone pre-balance (PREBAL) testing and meet EwE best practices (*56*).

### Stability metrics

The study presents a synthesis of diverse Ecopath models of marine ecosystems and an investigation of the impact of varying structural indicators on food web stability. Two categories of stability metrics were used to define stability, based on the assumption of small near-equilibrium disturbances and on alterations in biomass following huge deviations from equilibrium.

In the context of minor alterations, the ecosystem food web is characterized by local stability, as demonstrated by May’s quantitative method for stability. The magnitude of stability is represented by the negative real part of the maximum eigenvalue of the interaction strength matrix (*26*, *57*), local stability = −Re[λ(*A*)_max_]. Following the dynamical system stability criterion (*58*), the sign of the leading eigenvalue determines stability. Throughout this work, λ(*A*)_max_ denotes the eigenvalue with largest real part. Local stability requires Re[λ(*A*)_max_] < 0, where the negative sign is explicit. This expression has been widely used (*59*). The larger the value, the higher the stability of the food web

$$A=\left[\begin{array}{ccc} \begin{array}{cc} a_{11}-\lambda & a_{12}\\ a_{21} & a_{11}-\lambda \end{array} & \cdots & \begin{array}{c} a_{1n}\\ a_{12} \end{array}\\ \vdots \;\;\vdots & \ddots & \vdots \\ \begin{array}{cc} a_{n1}\; & a_{n2} \end{array} & \cdots & a_{\mathrm{nn}}-\lambda \end{array}\right]=0$$

where *a_ij_* refers to the strength of the effect of species *j* on species *i*. *a_ij_* = −[(*Q*/*B*)*_i_* × *DC_ij_*]/*B_i_*. Consequently, effect of the species *i* on the species *j* according to: *a_ji_* = −*e_ij_* × *a_ij_*, *e_ij_* is the efficiency with which *j* converts food into biomass, from feeding on *i*: *e_ij_* = (*P*/*B*)*_j_*/(*Q*/*B*)*_j_*. Solving this equation yields λ. Because of the diagonal element being 0, our local stability is actually relative local stability (called local stability).

In the context of huge disturbances, previous research has unequivocally demonstrated that stability is a multidimensional phenomenon (*60*–*62*). Stability may be quantified as resistance (*63*, *64*) or resilience (*65*, *66*). Resistance reflects the ability of an ecosystem to maintain its original state in the context of disturbances (also referred to as ecological resilience). Resilience is the speed with which an ecosystem returns to initial values after perturbations (also known as engineering resilience). Donohue *et al.* (*24*) reviewed the literature on stability-related issues and found that most studies were limited to one-dimensional quantification, which may lead to bias in the assessment of stability. Some progress has been made in the multidimensional stability of lakes (*40*), rock reefs (*39*), forest, and grassland ecosystems (*67*), yet the multidimensional characteristics of marine ecosystem stability have not been examined. In this study, two widely used stability metrics, namely, resistance and resilience, were quantified through the lens of external perturbation. To this end, the Ecosim software was used, whereby mortality rates were observed to increase by 5, 25, and 50% for each living group, respectively. Mortality rates reflect exogenous pollution, fishing, climate change, random factors, and other human activities working together with nature. The disturbance was maintained at a constant level for 5, 10, and 25 years, after which it decreased to its original value.

Resistance and resilience are expressed as (*30*, *31*)

$$\text{Resistance}=\frac{{\overset{´}{Y}}_{n}}{|\;Y_{e}-{\overset{´}{Y}}_{n}\;|}$$

$$\text{Resilience}=\frac{|\;Y_{e}-{\overset{´}{Y}}_{n}\;|}{|\;Y_{e+1}-{\overset{´}{Y}}_{n}\;|}$$

where ${\overset{´}{Y}}_{n}$ is the initial biomass before the disturbance. *Y_e_* is the maximum biomass after the disturbance. *Y*_*e*+1_ is the biomass after 1 year of disturbance cessation. High values correspond to higher stability. This measure of ecosystem stability, which is commonly used, is dimensionless, so direct comparisons can be made between studies, communities, and ecosystems with different levels of productivity. Furthermore, in light of the fact that the central parameters of Ecosim’s vulnerability affect the stability of the ecosystem (in terms of resilience and resistance), it was decided that the vulnerability values were randomized between [1,40] for each model.

### Ecosystem characteristics

The total number of interacting species in a food web, also defined as web size, has been used as a descriptor of web complexity due to its simplicity and effectiveness (*3*, *14*). The use of trophic species (living groups) rather than taxonomic species has become a widely accepted convention (*9*, *10*). The categorization of trophic species has been demonstrated to mitigate the dispersion of data and circumvent the redundancy of interactions, thereby reducing methodological bias in the dataset (*9*). Additional metrics that are frequently used to describe complexity include structural features of food webs, such as connectance and interactions. These metrics offer an intuitive explanation of the probability and strength of species interactions within the web (*21*). Furthermore, metrics pertaining to omnivory and energy cycling were considered, which delineate the structural characteristics of food webs and may potentially correlate with stability. These indicators were transformed logarithmically to satisfy the criteria of normal distribution. Comprehensive definitions and calculations of these indices are presented in table S3. These metrics have been extensively used in published EwE models to characterize the structural indicators of marine ecosystems (*68*).

### Statistical analysis

The primary scenario for data analysis and presentation of results was a moderate mortality rate (25%) with a moderate disturbance duration (10 years). Generalized multivariate linear mixed-effects models were used to assess the relationship between stability and all indicators. Variables that were statistically significant across multiple scenarios were included in the analysis of the final structural equation model, along with those that demonstrated diversity. Generalized linear mixed-effects models can handle both fixed and random effects. Specifically, we study the relationship between diversity, food web structure, and stability. However, each food web model comes from a different ecosystem, and differences between ecosystems can be taken into account, so ecosystems are set as random factors. Generalized linear mixed-effects models were used in conjunction with the piecewise structural equation model to elucidate the underlying mechanisms that govern marine ecosystem stability. The structural equation approach to data analysis allows for the simultaneous examination of direct and indirect causal relationships between multiple structures, offering a powerful tool for understanding complex systems. We started with a complete conceptual model (fig. S4) and followed a model simplification process in which we iteratively removed nonsignificant paths until only statistically significant paths remained or the model fitted better than further path removal results. To test the robustness of the structural equation model to perturbation magnitude and duration, analyses were conducted under nine separate scenarios. Following an analysis based on variance inflation factors, it was found that there was no multicollinearity among ecosystem indicators. Generalized linear mixed-effects models were analyzed using the “lme4” package in R, while structural equation models were analyzed using the “piecewiseSEM” package in R.

### Sensitivity analysis

It is our contention that the stability of an ecosystem cannot be attributed solely to the stability of different living groups, due to the varying proportions of biomass or contribution to the ecosystem. Accordingly, in the primary analysis, ecosystem stability was estimated as the median of community stability, which could circumvent the influence of outliers and effectively reflect the center of skewed data. To substantiate the reliability of the median, a series of stability indicators was calculated for the communities of each ecosystem. The stability indicator was taken at every 5% percentile in the range of 0 to 100% and incorporated into generalized multiple linear mixed-effects models.

To determine whether varying the intensity and duration of the disturbance would have an impact on the analysis of the structural equation model, the intensity (5, 25, and 50% mortality) and duration (5, 10, and 25 years) of the disturbance were varied, and a total of nine scenarios were simulated (fig. S5). The main text presents a simulated scenario with moderate mortality (25%) and a duration of 10 years.

### Food web resolution

The resolution of food webs can influence the estimation of taxa, connectivity, and interaction strength, which may introduce bias into the results. To evaluate the reliability of our analyses, we investigated the underlying mechanisms responsible for the stability of a set of best-resolved Ecopath models. We defined four levels of resolution and qualified one level for each taxon with the following indices: taxonomic species (e.g., Alaska Pollock, index = 1), family/class (e.g., Scorpaenidae and Nephropidae; index = 0.7), trophic function (e.g., small demersal fish; index = 0.4), and general name (e.g., phytoplankton and zooplankton; index = 0.1) (*13*). The resolution index of the Ecopath models is proportional to the mean resolution index of the living groups in each food web. We conducted a case study of the 29 best resolution models to investigate the stability-driving mechanisms and found results consistent with the overall analysis. Specifically, diversity improved stability with sparser connectance and weak ISI_sd_ (fig. S6).

### Nonlinear effect

The results of the random forest analysis indicated the potential for a nonlinear relationship between NLG and stability. To further investigate this, we used SEM to assess the possible nonlinear relationships. The findings suggested the existence of a nonlinear relationship, particularly within the resistance-driven pathway, where NLG was observed to directly promote resistance (fig. S7).

## Supplementary Materials

### This PDF file includes:

Supplementary Text Figs. S1 to S7 Tables S1 to S3 References
