# Supplementary material for: Mediating role of food web structure in linking diversity to multidimensional stability: Evidence from global marine ecosystems
Source: Sci Adv. 2025 Oct 10;11(41):eadv3841. doi: 10.1126/sciadv.adv3841 (PMC12513428; doi:10.1126/sciadv.adv3841)
Supplement: Supplementary file 1 — Supplementary Text Figs. S1 to S7 Tables S1 to S3 References [file sciadv.adv3841_sm.pdf]

Science Advances
AAAS

Supplementary Materials for

Mediating role of food web structure in linking diversity to multidimensional stability: Evidence from global marine ecosystems

Jianfeng Feng *et al.*

Corresponding author: Jianfeng Feng, fengjf@nankai.edu.cn; Nils Chr. Stenseth, n.c.stenseth@mn.uio.no

Sci. Adv. **11**, eadv3841 (2025)
DOI: 10.1126/sciadv.adv3841

This PDF file includes:

Supplementary Text
Figs. S1 to S7
Tables S1 to S3
References

Uncertainty analysis

EwE has several ways to deal with uncertainty, where uncertainty mainly consists of noise, parameter uncertainty, structural uncertainty, observation error, and implementation error. Among them, noise is random and cannot be captured completely. Structural uncertainty may arise from the way interactions are represented in the system or from external forcing factors that may or may not be recognized during model development. Observational errors include errors in sampling that can lead to uncertainty in biomass estimates. Few EwE models deal directly with observational error, though the effects of this uncertainty become evident in measures of uncertainty about parameter values derived by examining how much the parameter values can be changed without degrading the fit to the data. realization errors, on the other hand, arise in the evaluation of management strategies. Therefore, most EwE models focus on parameter uncertainty.

All Ecopath models uploaded to Ecobase have been analyzed for parametric uncertainty. This is an alternative method developed by EwE based on a parametric "pedigree". The assumption is that local data are more reliable than regional or global, and that guessed data are even more unreliable, but the most uncertain are the estimates from another model, especially those estimated by the Ecopath mass balance. The EwE spectrum is defined for the key Ecopath input parameters. Each parameter follows the logical categorization described above (from local data to model estimates).

Supplementary Figures

Fig. S1. Relationship between diversity, structure and stability quartiles of food web living groups.

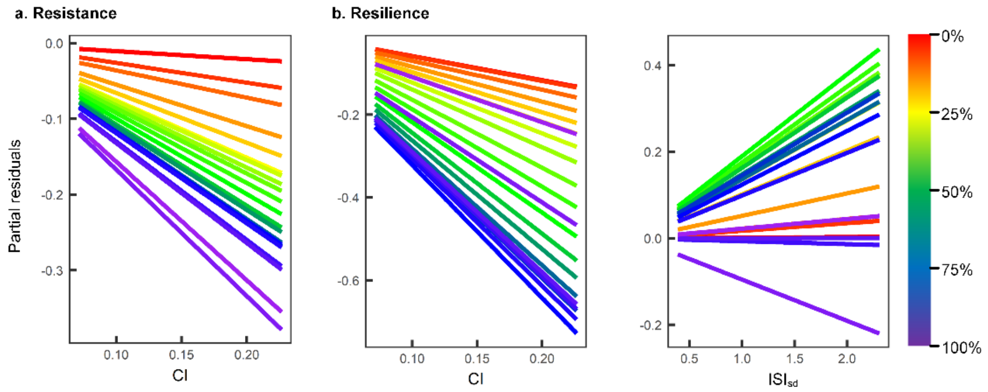

a, Linear relationship of resistance with different quartiles to CI. **b**, Linear relationship of resilience of different quartiles with CI and ISI_{sd}. CI: connectance index; ISI_{sd}: standard deviation of interaction strength index. All horizontal coordinates were log-transformed, and vertical coordinates were log-transformed partial residuals

Fig. S2. Pathways of diversity, food web structure and resistance in 9 scenarios.

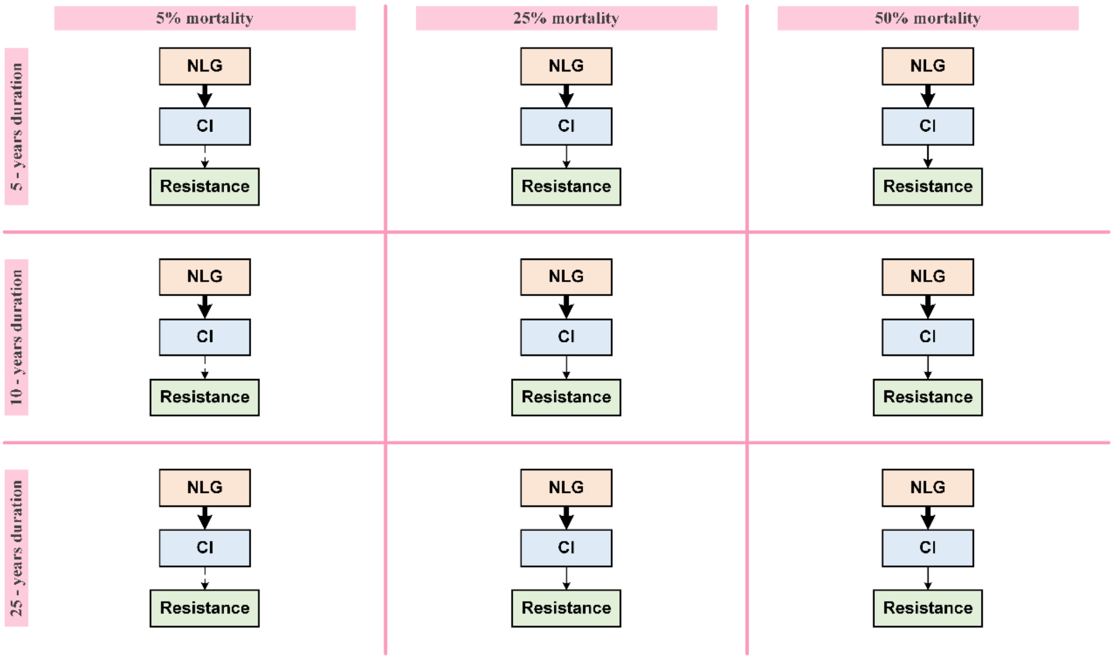

The same column indicates the same mortality disturbance. The same row indicates the same disturbance duration. NLG: number of living groups; CI: connectance index.

Fig. S3. Pathways of diversity, food web structure and resilience in 9 scenarios.

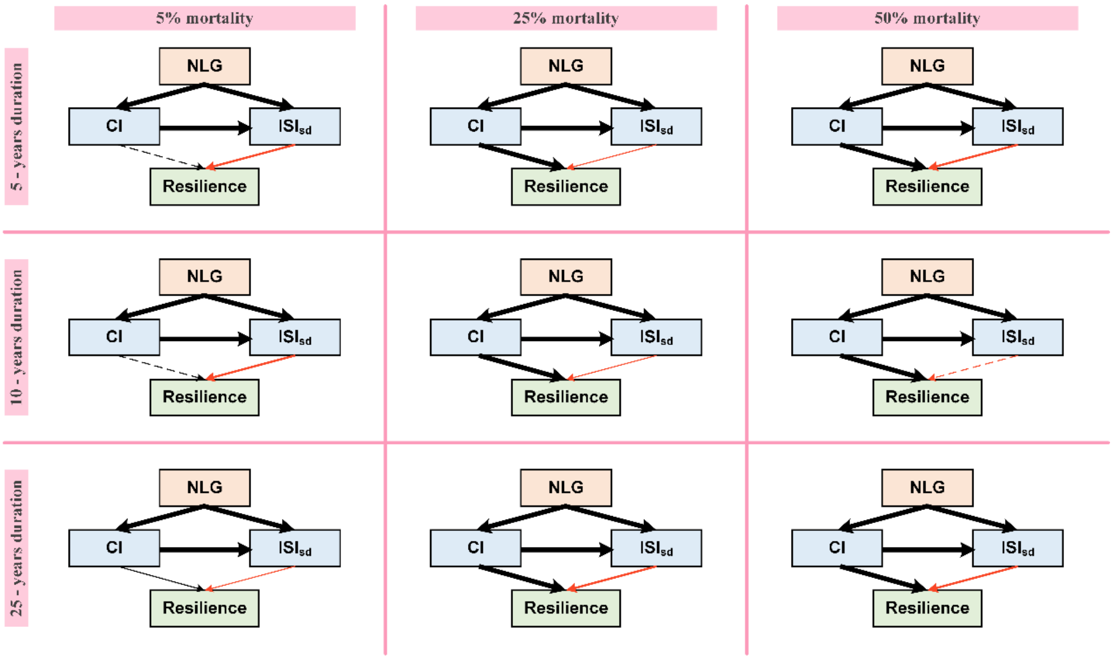

The same column indicates the same mortality disturbance. The same row indicates the same disturbance duration. NLG: number of living groups; CI: connectance index; ISI_{sd}: standard deviation of interaction strength index.

Fig. S4. Conceptual model of structural equation model.

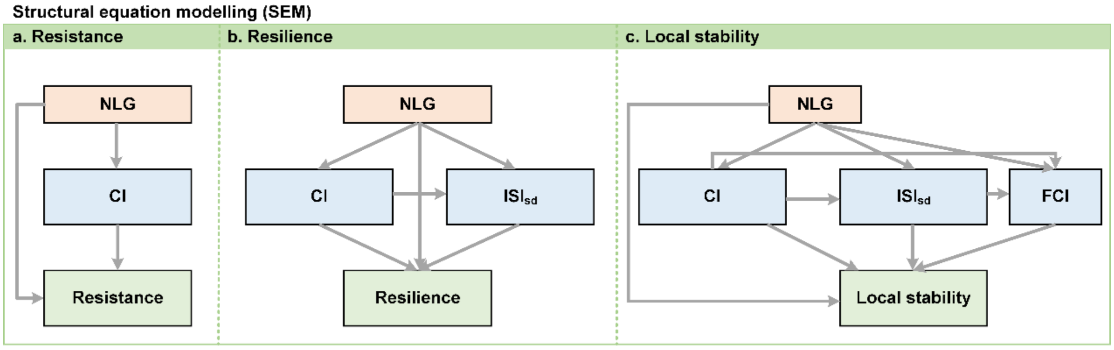

NLG: number of living groups; CI: connectance index; ISI$_{sd}$: standard deviation of interaction strength index; FCI: Finn's cycle index.

Fig. S5. Sensitivity analysis of the perturbations (mortality rate) under different intensity and duration.

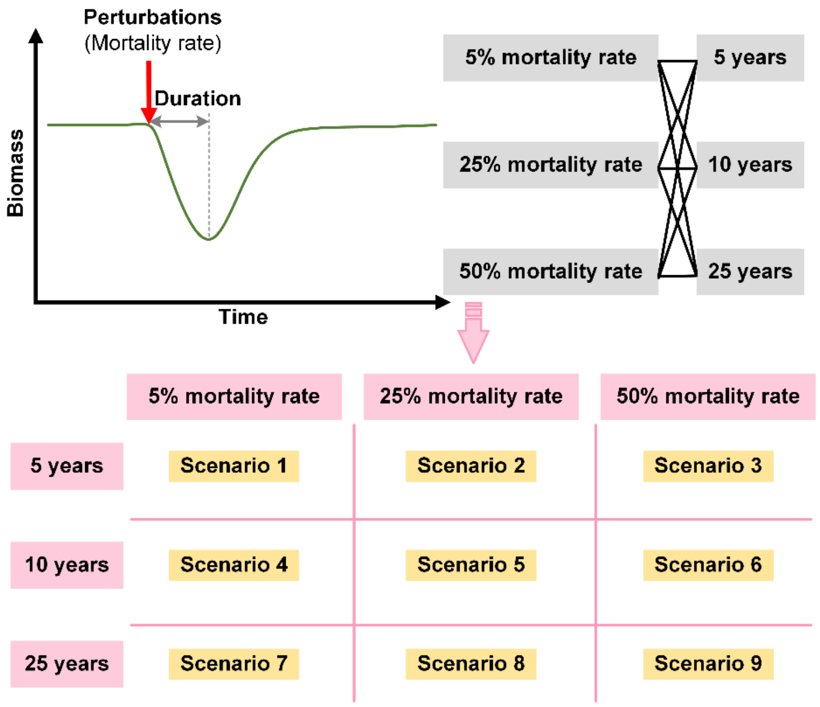

Fig. S6. Pathways of diversity driving food web structural features and altering stability, in the 29 best resolved food webs.

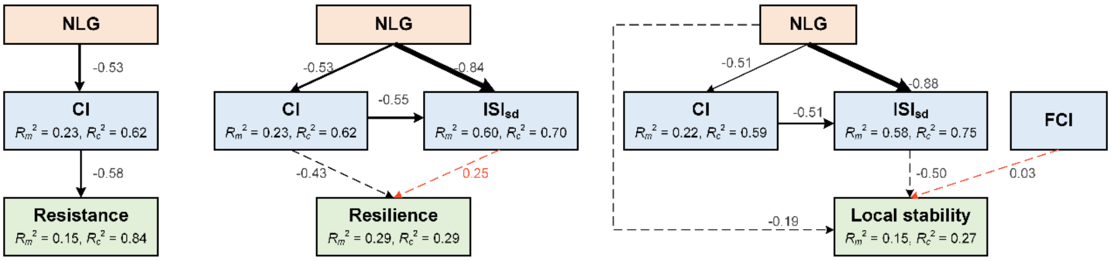

Gray and red arrows indicate negative and positive relationships, respectively. Dashed lines indicate insignificant paths. Gray and red arrows indicate negative and positive relationships, respectively. A unidirectional arrow indicates a causal relationship. Values associated with the arrows indicate standardized path coefficients. The size of the path indicates the magnitude of significance, with the thinnest, second largest, and thickest lines indicating significance at the 0.05, 0.01, and 0001 levels, respectively. R^2m indicates the proportion of each dependent variable that was explained by fixed effects. R^2c indicates the proportion of each dependent variable that was explained by both fixed and random effects. NLG: number of living groups; CI: connectance index; ISI_{sd}: standard deviation of interaction strength index; FCI: Finn's cycling index.

Fig. S7. Pathways of diversity driving food web structural features and altering stability, considering nonlinear paths with NLG.

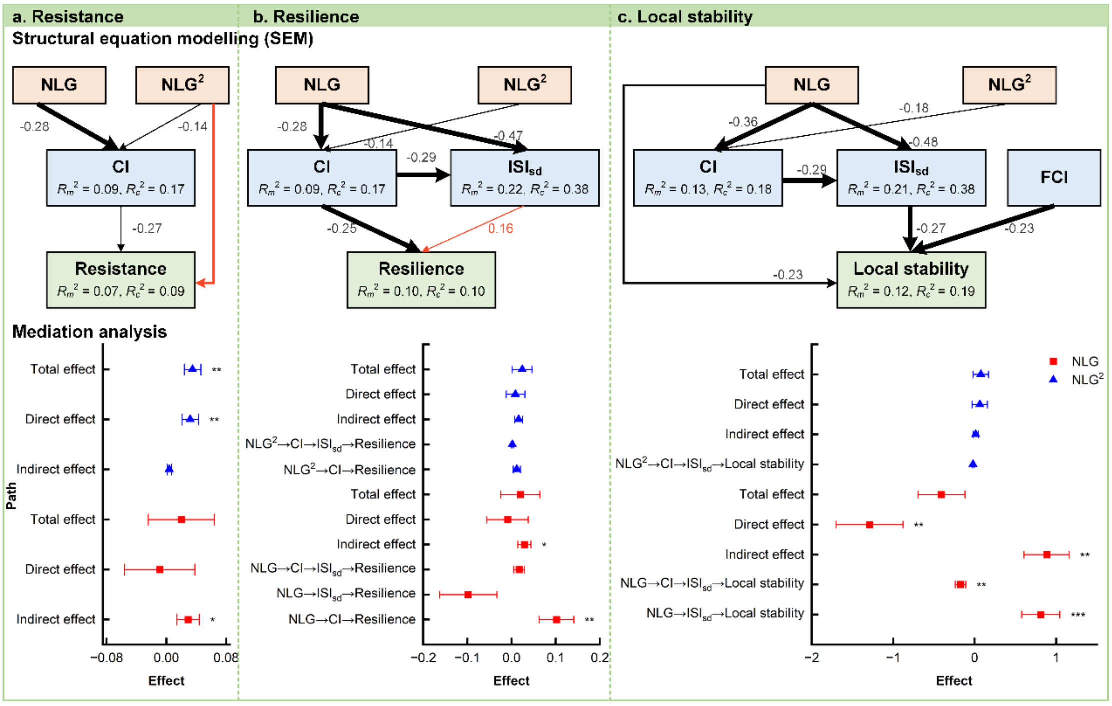

Only significant paths are shown. Gray and red arrows indicate negative and positive relationships, respectively. Gray and red arrows indicate negative and positive relationships, respectively. A unidirectional arrow indicates a causal relationship. Values associated with the arrows indicate standardized path coefficients. The size of the path indicates the magnitude of significance, with the thinnest, second largest, and thickest lines indicating significance at the 0.05, 0.01, and 0001 levels, respectively. $R^2{}_m$ indicates the proportion of each dependent variable that was explained by fixed effects. $R^2{}_c$ indicates the proportion of each dependent variable that was explained by both fixed and random effects. NLG: number of living groups; CI: connectance index; ISI_{sd}: standard deviation of interaction strength index; FCI: Finn's cycling index.

Supplementary Tables

Table S1 Marine food web models information. List of 217 marine food web models with habitats, modelled period, and references.

Model name	Ecosystem type	Year	Author and reference
Aegean Sea	Open ocean	2003 ~ 2007	Ioannis Keramidas(*69*)
Alaska, Prince William Sound	bay/fjord	1980 ~ 1989	Dalsgaard, J.(*70*)
Albatross Bay	bay/fjord	1986 ~ 1993	Okey, T.A.(*71*)
Aleutian Islands	continental shelf	1963 ~ 1963	Guénette, S.(*72*)
Alto Golfo de California	continental shelf		Morales-Zárate, M.V.(*73*)
Antarctic	open ocean	1970 ~ 1971	Hoover, C.
Apalachicola Bay	Estuary	2000 ~ 2050	Kira Allen(*74*)
Arachania	beach	1992 ~ 2007	Lercari, D.(*75*)
Australia North West Shelf	continental shelf	1986 ~ 1991	Bulman C.
Azores	Open ocean	1997 ~ 1998	Gomes, T.(*76*)
Azores archipelago	continental shelf	1997 ~ 1997	Guénette, S.(*77*)
Baie de Seine	Estuary	2000 ~ 2001	Ghassen Halouani(*78*)
Baja California	continental shelf	1970 ~ 1970	Cisneros-Montemayor, A.M.(*79*)
Bamboung	coastal lagoon	2006 ~ 2008	Colléter, M.(*80*)
Bamboung	coastal lagoon	2003 ~ 2003	Colléter, M.(*80*)
Barents Sea	continental shelf	1990 ~ 1990	Blanchard, J.L.(*81*)
Barnegat Bay	Estuary	1981 ~ 1982	Jim Vasslides(*82*)
Barra Del Chuy	beach	1992 ~ 2007	Lercari, D.(*75*)
Bay of Biscay	continental shelf	2013 ~ 2013	Fabien Moullec, Didier Gascuel(*83*)
Bay of Biscay	continental shelf	1998 ~ 1999	Ainsworth, C.H.(*84*)
Bay of Biscay	continental shelf	1970 ~ 1971	Ainsworth, C.H.(*84*)
Bay of Biscay	continental shelf	1994 ~ 2005	Lassalle, G.(*85*)
Bay of Biscay	continental shelf	1980 ~ 1980	Fabien Moullec, Didier Gascuel(*83*)
Black Sea	NA	1980 ~ 1981	Gucu, A.(*86*)
Black Sea	continental shelf	1960 ~ 1969	Akoglu, E.(*87*)
Black Sea	continental shelf	1990 ~ 1991	Gucu, A.(*86*)
Black Sea	continental shelf	1988 ~ 1994	Akoglu, E.(*87*)
Black Sea	continental shelf	1980 ~ 1987	Akoglu, E.(*87*)
BlackSea	continental shelf	1990 ~ 1991	Orek, H.
BlackSea	continental shelf	1955 ~ 1965	Gucu, A.(*86*)
Bolinao Coral Reef	coastal lagoon	1980 ~ 1981	Aliño, P.M.
British Columbia coast	continental shelf	1950 ~ 2000	Preikshot, D.B.(*88*)
Calvi Bay	bay/fjord	1998 ~ 1999	Pinnegar, J.K.(*89*)
Campeche	beach	1985 ~ 1990	Vega-Cendejas, M.E.(*77*)
Canada, Grand Banks of Newfoundland	open ocean	1985 ~ 1988	Bundy, A.(*90*)
Cap de Creus MPA - whole	Beach	2008 ~ 2012	Corrales, X.(*91*)
Cape Verde	continental shelf	1981 ~ 1985	Stobberup, K.A.
Caribbean	continental shelf	1980 ~ 1981	Melgo, J.L.(*92*)
Celestun	coastal lagoon		Chávez, E.A.(*77*)
Celestun mangrove	coastal lagoon	1992 ~ 1994	Vega-Cendejas, M.E.(*93*)
Celtic Sea	continental shelf	1985 ~ 2017	Pierre-Yves Hernvann(*94*)
Celtic Sea	continental shelf	2013 ~ 2013	Fabien Moullec, Didier Gascuel(*83*)
Celtic Sea	continental shelf	1980 ~ 1980	Fabien Moullec, Didier Gascuel(*83*)
Celtic Sea-Biscay	continental shelf	2012 ~ 2012	Bentorcha K,, Gascuel D., and Guénette S.(*95*)
Celtic Sea-Biscay	continental shelf	1980 ~ 1980	Bentorcha K., Gascuel D., and Guénette S.(*95*)
Central Atlantic	open ocean	1990 ~ 1991	Vasconcellos, M.(*96*)
Central Atlantic	open ocean	1950 ~ 1951	Vasconcellos, M.(*96*)
Central Baltic Sea	continental shelf	1974 ~ 1974	Tomczak et al.(*97*)
Central Chile	upwelling	1998 ~ 1999	Neira, S.(*98*)
Central Gulf of California	channel/strait	1978 ~ 1980	Arreguín-Sánchez, F.(*99*)
Cerbère-Banyuls MPA	Beach	2013 ~ 2014	Xavier Corrales, Daniel Vilas, Marta Coll(*91*)
Chantuto-Panzacola	Coastal lagoon		Jesús Manuel López Vila(*100*)
Chesapeake	bay/fjord	1950 ~ 1950	Christensen, V.(*101*)
Contemporary Alosine	Continental shelf	2000 ~ 2001	Dias(*102*)
Cyprus insular shelf trophic model	marine-coastal	2015 ~ 2017	Nikolas Michailidis(*103*)
Danajon Bank	coral reef	2010 ~ 2010	Regina Bacalso, FISH Project Philippines(*104*)
Deep Western Mediterranean sea	open ocean	2009 ~ 2010	Samuele Tecchio(*105*)
Denmark, Faroe Islands	open ocean	1997 ~ 1998	Zeller, D.(*106*)
East Bass Strait	continental shelf	1994 ~ 1994	Cathy Bulman
Eastern Bering Sea	open ocean	1979 ~ 1985	Trites, A.W.(*107*)

Eastern Corsican Coast	Continental shelf	2012 ~ 2014	Vanalderweireldt Lucie et al.(*108*)
Eastern Scotian Shelf	continental shelf	1980 ~ 1986	Bundy, A.
Eastern Scotian Shelf	continental shelf	1995 ~ 2000	Bundy, A.
Eastern Tropical Pacific	open ocean	1993 ~ 1997	Olson, R.J.(*109*)
Eritrea	coral reef	1998 ~ 1998	Tsehaye, I.(*110*)
Falkland Islands	open ocean	1990 ~ 1991	Cheung, W.W.L.
Florida Bay	Estuary	2006 ~ 2017	Mason Smith(*111*)
Galapagos	Coral reef	2006 ~ 2007	Diego J. Ruiz(*112*)
Galapagos, Floreana rocky reef	coral reef	2000 ~ 2001	Okey, T.A.(*113*)
Garonne	river	1990 ~ 1991	Palomares, M.L.D.(*77*)
Georges Bank	continental shelf	1996 ~ 2000	Link, S.(*114*)
Germany, Schlei Fjord	bay/fjord	1980 ~ 1981	Nauen, C.(*29*)
Gironde estuary	estuary	1991 ~ 1998	Lobry, J.
Golfo Dulce	bay/fjord	1993 ~ 1995	Wolff, M.(*115*)
Grand Banks of Newfoundland	open ocean	1900 ~ 1905	Heymans, S.J.J.(*90*)
Grand Banks of Newfoundland	open ocean	1980 ~ 1987	Heymans, S.J.J.(*116*)
Grand Banks of Newfoundland	open ocean	1990 ~ 1997	Heymans, S.J.J.(*116*)
Greenland, West Coast	open ocean	1997 ~ 1998	Pedersen, S.A.(*77*)
Guinea	open ocean	1998 ~ 1999	Guénette, S.(*96*)
Guinea	continental shelf	1985 ~ 1985	Gascuel, D.(*117*)
Guinea	continental shelf	2004 ~ 2004	Gascuel, D.(*117*)
Gulf of California	continental shelf	1990 ~ 2000	Lercari, D.(*118*)
Gulf of Carpentaria	bay/fjord	1990 ~ 1991	Okey, T.A.
Gulf of Gabes	Continental shelf	2000 ~ 2005	Tarek Hattab(*119*)
Gulf of Maine	continental shelf	1977 ~ 1987	Heymans, S.J.J.(*77*)
Gulf of Maine	continental shelf	1996 ~ 2000	Link, S.(*114*)
Gulf of Mexico	continental shelf	1950 ~ 1951	Walters, C.J.(*120*)
Gulf of Mexico	continental shelf	1980 ~ 1989	Browder, J.A.
Gulf of Nicoya	estuary	1993 ~ 1995	Wolff, M.(*121*)
Gulf of Salamanca	upwelling	1997 ~ 1998	Duarte, L.O.(*122*)
Gulf of Thailande	continental shelf	1963 ~ 1964	Christensen, V.(*123*)
Hudson Bay	bay/fjord	1970 ~ 1971	Hoover, C.(*124*)
Huizache-Caimanero	coastal lagoon	1984 ~ 1986	Zetina-Rejón, M.J.(*125*)
Humboldt Current	upwelling	1995 ~ 1996	J.Tam, M. Taylor(*126*)
Iceland	open ocean	1950 ~ 1951	Buchary, E.A.(*77*)
Icelandic shelf	open ocean	1997 ~ 1998	Samb, B.
Independence Bay	bay/fjord	1996 ~ 1997	Taylor, M.H.(*127*)
Irish Sea	continental shelf	1973 ~ 1974	Lees, K.(*128*)
Jalisco and Colima Coast	continental shelf	1995 ~ 1996	Galván-Piña, V.H.
Jurien Bay	coastal lagoon	2007 ~ 2008	Lozano-Montes, H.M.(*129*)
Kaloko Honokohau	coral reef	2005 ~ 2005	Wabnitz, C.C.(*130*)
Kuosheng Bay	bay/fjord	1998 ~ 2001	Lin, H.-J.(*131*)
Lagoon Chiku - Taiwan	coastal lagoon	1997 ~ 1998	Lin, H.-J.(*132*)
Lesser Antilles	open ocean	2001 ~ 2005	Mohammed, E.
Liberia	continental shelf	2005 ~ 2006	Kay, D.W.
Looe Key National Marine Sanctuary	coral reef	1980 ~ 1989	McCormick Venier, J.(*133*)
Low Barents sea	continental shelf	1995 ~ 1996	Blanchard, J.L.(*81*)
Malangen Fjord	Bay/fjord	2017 ~ 2018	Daniel Vilas(*134*)
Marmara Sea 1990s	Continental shelf	1995 ~ 1996	Saygu et al. (2023)(*135*)
Marmara Sea 2000s	Continental shelf	2005 ~ 2006	Saygu et al. (2023)(*135*)
Marmara Sea 2010s	Continental shelf	2015 ~ 2016	Saygu et al. (2023)(*135*)
Mauritania	open ocean	1998 ~ 1999	Ould Taleb Ould Sidi, M.M.(*96*)
Mauritania	open ocean	1987 ~ 1988	Ould Taleb Ould Sidi, M.M.(*96*)
Mauritanie	continental shelf	1991 ~ 1991	Sylvie Guenette and Beyah Meissa(*136*)
Medes Island MPA	Beach	2000 ~ 2004	Xavier Corrales(*91*)
Mid-Atlantic Bight	continental shelf	1996 ~ 2000	Link, S.(*114*)
Miramare	bay/fjord	2000 ~ 2003	Libralato, S.(*137*)
Moreton Bay Ecosystem	bay/fjord	1990 ~ 2014	Esther Fondo(*138*)
Morocco	open ocean	1985 ~ 1987	Stanford, R.(*77*)
Mount St Michel Bay	bay/fjord	2003 ~ 2004	Le Pape Olivier(*139*)
Narragansett Bay food web	Estuary	1994 ~ 2019	Austin Humphries(*140*)
New Foundland	open ocean	1985 ~ 1986	Ainsworth, C.H.(*141*)
Ningaloo	coral reef	2007 ~ 2007	Beth Fulton
North Aegean	continental shelf	2003 ~ 2006	Tsagarakis K.(*142*)
North Atlantic	open ocean	1950 ~ 1951	Vasconcellos, M.
North Atlantic	open ocean	1997 ~ 1998	Vasconcellos, M.
North Benguela	upwelling	1600 ~ 1601	Watermeyer, K.(*143*)
North Benguela	upwelling	1900 ~ 1901	Watermeyer, K.(*143*)

North Benguela	upwelling	1967 ~ 1968	Watermeyer, K.(*143*)
North Benguela	upwelling	1990 ~ 1991	Watermeyer, K.(*143*)
North Brazil	estuary	1970 ~ 1990	Wolff, M.(*144*)
North East Pacific	continental shelf	1950 ~ 1950	Preikshot, D.B.(*88*)
Northern Benguela	upwelling	1956 ~ 1957	Heymans, S.J.J.
Northern British Columbia	channel/strait	1950 ~ 1951	Ainsworth, C.H.(*90*)
Northern British Columbia	channel/strait	2000 ~ 2001	Ainsworth, C.H.(*90*)
Northern Californian Current	upwelling	1990 ~ 2000	Field, J.C.(*145*)
Northern Californian Current	upwelling	1960 ~ 1969	Walters, C.J.(*145*)
Northern Gulf of Mexico	coastal lagoon	2005 ~ 2009	Skyler Sagarese(*146*)
Northern Gulf of St Lawrence	channel/strait	1990 ~ 1991	Savenkoff, C.(*147*)
Northern Gulf St Lawrence	estuary	1985 ~ 1987	Morissette, L.(*148*)
Northern Humboldt Current	upwelling	1997 ~ 1998	J.Tam, M. Taylor(*126*)
North Sea	continental shelf	1981 ~ 1982	Christensen, V.
North Sea	continental shelf	1974 ~ 1975	Christensen, V.
North Sea	continental shelf	1991 ~ 1992	Mackinson, S.(*149*)
North South of China Sea	continental shelf	1970 ~ 1971	Cheung, W.W.L.(*150*)
Northwest Africa	open ocean	1987 ~ 1987	Morissette, L.(*151*)
Orbetello Lagoon	coastal lagoon	1996 ~ 1997	Brando, V.E.(*152*)
Pagasitikos Gulf	bay/fjord	2008 ~ 2008	Donna Dimarchopoulou(*153*)
Paraná River Floodplain	river	1992 ~ 1995	Angelini, R.(*154*)
Peru	upwelling	1973 ~ 1979	Jarre-Teichmann, A.
Peru	upwelling	1953 ~ 1953	Guénette, S.(*155*)
Peru	upwelling	1953 ~ 1959	Jarre-Teichmann, A.
Peru	upwelling	1960 ~ 1969	Jarre-Teichmann, A.
Porsangerfjord Area 3	Bay/fjord		Torstein Pedersen(*156*)
Port Cros	continental shelf	1998 ~ 2008	Valls, A.(*157*)
Portofino	coastal lagoon	2007 ~ 2014	Giulia PRATO(*158*)
Port Phillip Bay	bay/fjord	1994 ~ 1995	Fulton, E.A.(*159*)
Prince William Sound	bay/fjord	1994 ~ 1996	Okey, T.A.(*160*)
Raja Ampat	coral reef	1990 ~ 1991	Ainsworth, C.H.(*161*)
Raja Ampat	coral reef	2005 ~ 2006	Ainsworth, C.H.(*161*)
Restored Alosine Biomass	Continental shelf	2000 ~ 2001	Dias(*102*)
Ria Formosa	coastal lagoon	1996 ~ 1997	Gamito, S.(*162*)
Ria-Lake Tapajos	River	2013 ~ 2044	Leonardo Capitani(*163*)
Rocas Atoll	Coral reef	2012 ~ 2019	Leonardo Capitani(*163*)
Santa Pola Bay	Fish farm	2001 ~ 2007	Bayle-Sempere, J.T.(*164*)
Seagrass and Mangrove Terminos Lagoon	coastal lagoon	1990 ~ 2000	Rivera-Arriaga, E.(*165*)
Sechura Bay	bay/fjord	1996 ~ 1997	Taylor, M.H.(*166*)
Senegambia	continental shelf	1990 ~ 1991	Samb, B.(*167*)
Sierra Leone	continental shelf	1964 ~ 1965	Heymans, S.J.J.(*167*)
Sierra Leone	continental shelf	1990 ~ 1991	Heymans, S.J.J.(*167*)
Sierra Leone 1978	continental shelf	1978 ~ 1979	Heymans, S.J.J.(*167*)
Sinaloa sur MEXICO	continental shelf	1994 ~ 1997	Salcido-Guavara, L.A.
Sirinhaém River	Estuary	2013 ~ 2015	Lira et al.(*168*)
Sítios Novos reservoir	Reservoir	2011 ~ 2012	Bezerra, LAV(*169*)
Sonda_Campeche_Act	continental shelf	1988 ~ 1994	Zetina-Rejón, M.J.(*170*)
Sonda de Campeche	continental shelf	1988 ~ 1994	Manickchand-Heileman, S.(*171*)
Sørfjord	bay/fjord	1993 ~ 1996	Falk-Petersen, J.(*172*)
South Benguela	upwelling	1900 ~ 1901	Watermeyer, K.(*143*)
South Benguela	upwelling	1978 ~ 1979	Shannon, L.(*173*)
South Benguela	upwelling	1600 ~ 1601	Watermeyer, K.(*143*)
South East Alaska	continental shelf	1963 ~ 1963	Guénette, S.(*72*)
Southern Brazil	continental shelf	1980 ~ 1981	Vasconcellos, M.(*174*)
Southern Gulf of St. Lawrence	continental shelf	1980 ~ 1981	Savenkoff, C.(*146*)
Southern New England	continental shelf	1996 ~ 2000	Link, S.(*114*)
South of Benguela	upwelling	1960 ~ 1961	Watermeyer, K.(*143*)
South Shetlands	open ocean	1990 ~ 2000	Bredesen, E.L.(*175*)
South western Gulf of Mexico	continental shelf	1970 ~ 1980	Arreguín-Sánchez, F.(*176*)
South West Viet nam	continental shelf	1994 ~ 1995	Christensen, V.(*177*)
Sri Lanka	continental shelf	2000 ~ 2001	Haputhantri, S.S.K.(*178*)
Strait of Georgia	channel/strait	1950 ~ 1950	Preikshot, D.B.(*88*)
Strait of Gergia	channel/strait	1950 ~ 1951	Martell, S.J.D.(*179*)
Tamiahua	coastal lagoon	1989 ~ 1989	Abarca-Arenas, L.G.
Tampa Bay	bay/fjord	1950 ~ 2004	Walters, C.J.(*120*)
Tampa Bay	Estuary	2005 ~ 2010	Chagaris, David
Tasmanian Seamounts Marine Reserve	open ocean	1992 ~ 1996	Cathy Bulman

Tasmanian waters	continental shelf	1993 ~ 2007	Gabrielle Nowara and Reg Watson(*180*)
Terminos Lagoon	coastal lagoon	1980 ~ 1990	Manickchand-Heileman, S.(*181*)
Thau	coastal lagoon	1980 ~ 1989	Palomares, M.L.D.
Thermaikos Gulf	bay/fjord	1998 ~ 2000	Donna Dimarchopoulou(*182*)
USA, Mid Atlantic Bight	continental shelf	1995 ~ 1998	Okey, T.A.(*77*)
USA, South Atlantic Continental Shelf	open ocean	1995 ~ 1998	Okey, T.A.(*77*)
Venezuela shelf	continental shelf	1980 ~ 1989	Mendoza, J.J.
Virgin Islands	coral reef	1960 ~ 1999	Opitz, S.(*183*)
West Baffin Bay, Coastal and Shelf	Bay/fjord	2016 ~ 2017	Sara Pedro(*184*)
West Coast of Peninbsular Malaysia	continental shelf	1972 ~ 1973	Christensen, V.(*177*)
West coast of Sabah	continental shelf	1972 ~ 1973	Garces, L.R.(*185*)
West Coast Vancouver Island	continental shelf	1950 ~ 1950	Martell, S.J.D.(*186*)
Western Antarctic Peninsula	Continental shelf	1996 ~ 1997	Dahood, A(*187*)
Western Bering Sea	continental shelf	1981 ~ 1990	Aydin, K.Y.(*188*)
Western Channel	channel/strait	1993 ~ 1994	Araújo, J.N.
Western Channel	channel/strait	1973 ~ 1973	Araújo, J.N.
Western Tropical Pacific Ocean	open ocean	1990 ~ 2001	Godinot, O.(*189*)
West Florida Shelf	continental shelf	1985 ~ 2018	David Chagaris
West Florida Shelf Historic Model	Continental shelf	1950 ~ 2010	Chagaris, David(*190*)
West Scotland	continental shelf	2000 ~ 2004	Morissette, L.(*191*)
West scotland DeepSea	open ocean	1974 ~ 1975	Heymans, S.J.J.
Yucatan	continental shelf	1987 ~ 1988	Arreguín-Sánchez, F.(*192*)

Table S2 Basic input parameters for Ecopath model.

Input parameter	Name	Unit
B	Biomass	t/km^2
P/B	Production/biomass ratio	/year
Q/B	Consumption/biomass ratio	/year
EE	Ecotrophic efficiency	(proportion)
BA	Biomass accumulation	t/km^{2}year
E	Net migration	t/km^{2}year
DC_{ij}	Proportion of prey i in diet of predator j	(proportion)
Y	Catches by fleet	t/km^{2}year

Table S3 List of food web indicators with acronyms, descriptions, computational formulas.

Name	Acronym	Description	Formula
Number of living groups	NLG	Total number of function groups in a system	
Connectance index	CI	The ratio of the number of actual links to the number of possible links.	$CI = \frac{L}{N^2}$ *L* is the actual number of links in a food web; *N* is the number of all function groups.
Standard deviation of interaction strength index	ISI_{sd}	The standard deviation of all elements of the community (Jacobian) matrix.	$a_{ij} = -\frac{\left(\left(\frac{Q}{B}\right)_i \times DC_{ij}\right)}{B_i}$
Mean of interaction strength index	ISI_{mean}	The mean value of all elements of the community (Jacobian) matrix.	$a_{ji} = -e_{ij} \times a_{ij}$ $e_{ij} = \frac{\left(\frac{P}{B}\right)_j}{\left(\frac{Q}{B}\right)_j}$
System omnivory index	SOI	Weighted average of trophic level variance of predatory trophic groups among consumers.	$OI_i = \sum_{j=1}^{n} \left(TL_j - (TL_i - 1)\right)^2 \times DC_{ij}$ $SOI = \frac{\sum_{j=1}^{n} OI_i \times \log Q_i}{\sum_{j=1}^{n} \log Q_i}$ TL_i and TL_j is the trophic levels of prey *i* and predator *j*. Qi is the food intake by predators. *DCij* is the food composition.
Predatory cycle index	PCI	The ratio of an ecosystem's throughput that is recycled by excluding the cycling through detritus.	$PCI = \frac{TST_{no\ det}}{TST}$ $TST_{no\ det}$ is the system throughput that is recycled by excluding the cycling through detritus. TST is the total system throughput.
Finn's cycling index	FCI	The ratio of an ecosystem's throughput that is recycled.	$FCI = \frac{TST_c}{TST}$ TST_c is the total system throughput that is recycled.
Finn's mean path length	FML	Number of trophic groups whose energy flows.	$FCI = \frac{TST}{\sum TE + \sum TR}$ *TE* is the food web energy output and *TR* is the respiration.

REFERENCES AND NOTES

1. M. Loreau, C. De Mazancourt, Biodiversity and ecosystem stability: A synthesis of underlying mechanisms. *Ecol. Lett.* **16**, 106–115 (2013).

2. B. Worm, J. E. Duffy, Biodiversity, productivity and stability in real food webs. *Trends Ecol. Evol.* **18**, 628–632 (2003).

3. R. MacArthur, Fluctuations of animal populations and a measure of community stability. *Ecology* **36**, 533–536 (1955).

4. C. S. Elton, *The Ecology of Invasions by Animals and Plants* (Springer International Publishing, 1958); http://link.springer.com/10.1007/978-3-030-34721-5.

5. R. M. May, Will a large complex system be stable? *Nature* **238**, 413–414 (1972).

6. S. Allesina, S. Tang, The stability–complexity relationship at age 40: A random matrix perspective. *Popul. Ecol.* **57**, 63–75 (2015).

7. J. E. Cohen, C. M. Newman, The stability of large random matrices and their products. *Ann. Probab.* **12**, 283–310 (1984).

8. J. A. Dunne, "The network structure of food webs" in *Ecological Networks*, M. Pascual, J. A. Dunne, Eds. (Oxford Univ. Press, 2005), pp. 27–92.

9. S. L. Pimm, J. H. Lawton, J. E. Cohen, Food web patterns and their consequences. *Nature* **350**, 669–674 (1991).

10. J. A. Dunne, R. J. Williams, N. D. Martinez, Food-web structure and network theory: The role of connectance and size. *Proc. Natl. Acad. Sci. U.S.A.* **99**, 12917–12922 (2002).

11. S. Allesina, S. Tang, Stability criteria for complex ecosystems. *Nature* **483**, 205–208 (2012).

12. T. Gross, L. Rudolf, S. A. Levin, U. Dieckmann, Generalized models reveal stabilizing factors in food webs. *Science* **325**, 747–750 (2009).

13. C. Jacquet, C. Moritz, L. Morissette, P. Legagneux, F. Massol, P. Archambault, D. Gravel, No complexity–stability relationship in empirical ecosystems. *Nat. Commun.* **7**, 12573 (2016).

14. S. L. Pimm, Complexity and stability: Another look at MacArthur's original hypothesis. *Oikos* **33**, 351 (1979).

15. X. Chen, J. E. Cohen, Global stability, local stability and permanence in model food webs. *J. Theor. Biol.* **212**, 223–235 (2001).

16. J. A. Dunne, R. J. Williams, Cascading extinctions and community collapse in model food webs. *Philos. Trans. R. Soc. B* **364**, 1711–1723 (2009).

17. D. T. Haydon, Maximally stable model ecosystems can be highly connected. *Ecology* **81**, 2631–2636 (2000).

18. K. McCann, A. Hastings, G. R. Huxel, Weak trophic interactions and the balance of nature. *Nature* **395**, 794–798 (1998).

19. W. F. Fagan, Omnivory as a stabilizing feature of natural communities. *Am. Nat.* **150**, 554–567 (1997).

20. C. F. Jordan, J. R. Kline, D. S. Sasscer, Relative stability of mineral cycles in forest ecosystems. *Am. Nat.* **106**, 237–253 (1972).

21. P. Landi, H. O. Minoarivelo, Å. Brännström, C. Hui, U. Dieckmann, Complexity and stability of ecological networks: A review of the theory. *Popul. Ecol.* **60**, 319–345 (2018).

22. J. E. Cohen, F. Briand, Trophic links of community food webs. *Proc. Natl. Acad. Sci. U.S.A.* **81**, 4105–4109 (1984).

23. Y. Yonatan, G. Amit, J. Friedman, A. Bashan, Complexity–stability trade-off in empirical microbial ecosystems. *Nat. Ecol. Evol.* **6**, 693–700 (2022).

24. I. Donohue, H. Hillebrand, J. M. Montoya, O. L. Petchey, S. L. Pimm, M. S. Fowler, K. Healy, A. L. Jackson, M. Lurgi, D. McClean, N. E. O'Connor, E. J. O'Gorman, Q. Yang, Navigating the complexity of ecological stability. *Ecol. Lett.* **19**, 1172–1185 (2016).

25. E. Thébault, C. Fontaine, Stability of ecological communities and the architecture of mutualistic and trophic networks. *Science* **329**, 853–856 (2010).

26. S. L. Pimm, J. H. Lawton, Number of trophic levels in ecological communities. *Nature* **268**, 329–331 (1977).

27. Y. Hautier, E. W. Seabloom, E. T. Borer, P. B. Adler, W. S. Harpole, H. Hillebrand, E. M. Lind, A. S. MacDougall, C. J. Stevens, J. D. Bakker, Y. M. Buckley, C. Chu, S. L. Collins, P. Daleo, E. I. Damschen, K. F. Davies, P. A. Fay, J. Firn, D. S. Gruner, V. L. Jin, J. A. Klein, J. M. H. Knops, K. J. La Pierre, W. Li, R. L. McCulley, B. A. Melbourne, J. L. Moore, L. R. O'Halloran, S. M. Prober, A. C. Risch, M. Sankaran, M. Schuetz, A. Hector, Eutrophication weakens stabilizing effects of diversity in natural grasslands. *Nature* **508**, 521–525 (2014).

28. C. De Mazancourt, F. Isbell, A. Larocque, F. Berendse, E. De Luca, J. B. Grace, B. Haegeman, H. Wayne Polley, C. Roscher, B. Schmid, D. Tilman, J. Van Ruijven, A. Weigelt, B. J. Wilsey, M. Loreau, Predicting ecosystem stability from community composition and biodiversity. *Ecol. Lett.* **16**, 617–625 (2013).

29. V. Christensen, D. Pauly, ECOPATH II—A software for balancing steady-state ecosystem models and calculating network characteristics. *Ecol. Model.* **61**, 169–185 (1992).

30. K. Huang, J. Xia, High ecosystem stability of evergreen broadleaf forests under severe droughts. *Glob. Chang. Biol.* **25**, 3494–3503 (2019).

31. F. Isbell, D. Craven, J. Connolly, M. Loreau, B. Schmid, C. Beierkuhnlein, T. M. Bezemer, C. Bonin, H. Bruelheide, E. De Luca, A. Ebeling, J. N. Griffin, Q. Guo, Y. Hautier, A. Hector, A. Jentsch, J. Kreyling, V. Lanta, P. Manning, S. T. Meyer, A. S. Mori, S. Naeem, P. A. Niklaus, H. W. Polley, P. B. Reich, C. Roscher, E. W. Seabloom, M. D. Smith, M. P. Thakur, D. Tilman, B. F. Tracy, W. H. Van Der Putten, J. Van Ruijven, A. Weigelt, W. W. Weisser, B.

Wilsey, N. Eisenhauer, Biodiversity increases the resistance of ecosystem productivity to climate extremes. *Nature* **526**, 574–577 (2015).

32. S. Nie, J. Zheng, M. Luo, M. Loreau, D. Gravel, S. Wang, Will a large complex system be productive? *Ecol. Lett.* **26**, 1325–1335 (2023).

33. D. M. Busiello, S. Suweis, J. Hidalgo, A. Maritan, Explorability and the origin of network sparsity in living systems. *Sci. Rep.* **7**, 12323 (2017).

34. A. J. Wright, A. Ebeling, H. De Kroon, C. Roscher, A. Weigelt, N. Buchmann, T. Buchmann, C. Fischer, N. Hacker, A. Hildebrandt, S. Leimer, L. Mommer, Y. Oelmann, S. Scheu, K. Steinauer, T. Strecker, W. Weisser, W. Wilcke, N. Eisenhauer, Flooding disturbances increase resource availability and productivity but reduce stability in diverse plant communities. *Nat. Commun.* **6**, 6092 (2015).

35. D. Mateos-Molina, I. Bejarano, S. J. Pittman, M. Möller, M. Antonopoulou, R. W. Jabado, Coastal lagoons in the United Arab Emirates serve as critical habitats for globally threatened marine megafauna. *Mar. Pollut. Bull.* **200**, 116117 (2024).

36. S. O. Grose, L. Pendleton, A. Leathers, A. Cornish, S. Waitai, Climate change will re-draw the map for Marine Megafauna and the people who depend on them. *Front. Mar. Sci.* **7**, 547 (2020).

37. H. Hamaoka, A. Kaneda, N. Okuda, K. Omori, Upwelling-like bottom intrusion enhances the pelagic–benthic coupling by a fish predator in a coastal food web. *Aquat Ecol* **48**, 63–71 (2014).

38. S. A. Thompson, W. J. Sydeman, J. A. Santora, B. A. Black, R. M. Suryan, J. Calambokidis, W. T. Peterson, S. J. Bograd, Linking predators to seasonality of upwelling: Using food web indicators and path analysis to infer trophic connections. *Prog. Oceanogr.* **101**, 106–120 (2012).

39. I. Donohue, O. L. Petchey, J. M. Montoya, A. L. Jackson, L. McNally, M. Viana, K. Healy, M. Lurgi, N. E. O'Connor, M. C. Emmerson, On the dimensionality of ecological stability. *Ecol. Lett.* **16**, 421–429 (2013).

40. H. Hillebrand, S. Langenheder, K. Lebret, E. Lindström, Ö. Östman, M. Striebel, Decomposing multiple dimensions of stability in global change experiments. *Ecol. Lett.* **21**, 21–30 (2018).

41. S. M. Vallina, C. Le Quéré, Stability of complex food webs: Resilience, resistance and the average interaction strength. *J. Theor. Biol.* **272**, 160–173 (2011).

42. J. Wu, S. Liang, Assessing terrestrial ecosystem resilience using satellite leaf area index. *Remote Sens* **12**, 595 (2020).

43. D. L. Hoover, A. K. Knapp, M. D. Smith, Resistance and resilience of a grassland ecosystem to climate extremes. *Ecology* **95**, 2646–2656 (2014).

44. R. T. Paine, Food-web analysis through field measurement of per capita interaction strength. *Nature* **355**, 73–75 (1992).

45. C. Van Altena, L. Hemerik, P. C. De Ruiter, Food web stability and weighted connectance: The complexity-stability debate revisited. *Theor Ecol* **9**, 49–58 (2016).

46. C. Borrvall, B. Ebenman, T. J. Tomas Jonsson, Biodiversity lessens the risk of cascading extinction in model food webs. *Ecol. Lett.* **3**, 131–136 (2000).

47. A. Bodini, C. Bondavalli, Towards a sustainable use of water resources: A whole-ecosystem approach using network analysis. *IJEP* **18**, 463 (2002).

48. S. Manickchand-Heileman, J. Mendoza-Hill, A. L. Kong, F. Arocha, A trophic model for exploring possible ecosystem impacts of fishing in the Gulf of Paria, between Venezuela and Trinidad. *Ecol. Model.* **172**, 307–322 (2004).

49. R. E. Ulanowicz, “Community measures of marine food networks and their possible applications” in *Flows of Energy and Materials in Marine Ecosystems*, M. J. R. Fasham, Ed. (Springer US, 1984), pp. 23–47.

50. R. E. Ulanowicz, Quantitative methods for ecological network analysis. *Comput. Biol. Chem.* **28**, 321–339 (2004).

51. S. Villasante, F. Arreguín-Sánchez, J. J. Heymans, S. Libralato, C. Piroddi, V. Christensen, M. Coll, Modelling marine ecosystems using the Ecopath with Ecosim food web approach: New insights to address complex dynamics after 30 years of developments. *Ecol. Model.* **331**, 1–4 (2016).

52. J. W. Bentley, N. Serpetti, C. J. Fox, J. J. Heymans, D. G. Reid, Retrospective analysis of the influence of environmental drivers on commercial stocks and fishing opportunities in the Irish Sea. *Fish. Oceanogr.* **29**, 415–435 (2020).

53. M. Colléter, A. Valls, J. Guitton, D. Gascuel, D. Pauly, V. Christensen, Global overview of the applications of the Ecopath with Ecosim modeling approach using the EcoBase models repository. *Ecol. Model.* **302**, 42–53 (2015).

54. M. Colléter, A. Valls, J. Guitton, M. Lyne, F. A.- Sánchez, V. Christensen, D. D. Gascuel, D. Pauly, “EcoBase: A repository solution to gather and communicate information from EwE models,” thesis, Fisheries Centre, University of British Columbia, Canada (2013).

55. J. Zheng, U. Brose, D. Gravel, B. Gauzens, M. Luo, S. Wang, Asymmetric foraging lowers the trophic level and omnivory in natural food webs. *J. Anim. Ecol.* **90**, 1444–1454 (2021).

56. J. J. Heymans, M. Coll, J. S. Link, S. Mackinson, J. Steenbeek, C. Walters, V. Christensen, Best practice in Ecopath with Ecosim food-web models for ecosystem-based management. *Ecol. Model.* **331**, 173–184 (2016).

57. M. Emmerson, J. M. Yearsley, Weak interactions, omnivory and emergent food-web properties. *Proc. R. Soc. Lond. B* **271**, 397–405 (2004).

58. S. Strogatz, *Nonlinear Dynamics and Chaos: With Applications to Physics*, *Biology*, *Chemistry and Engineering*. Studies in nonlinearity (CRC Press, 2nd ed., 2018).

59. M. G. Neubert, H. Caswell, Alternatives to resilience for measuring the responses of ecological systems to perturbations. *Ecology* **78**, 653–665 (1997).

60. S. L. Pimm, The complexity and stability of ecosystems. *Nature* **307**, 321–326 (1984).

61. G. H. Orians, "Diversity, stability and maturity in natural ecosystems" in *Unifying Concepts in Ecology*, W. H. Van Dobben, R. H. Lowe-McConnell, Eds. (Springer Netherlands, 1975), pp. 139–150.

62. V. Domínguez-García, V. Dakos, S. Kéfi, Unveiling dimensions of stability in complex ecological networks. *Proc. Natl. Acad. Sci. U.S.A.* **116**, 25714–25720 (2019).

63. F. Pennekamp, M. Pontarp, A. Tabi, F. Altermatt, R. Alther, Y. Choffat, E. A. Fronhofer, P. Ganesanandamoorthy, A. Garnier, J. I. Griffiths, S. Greene, K. Horgan, T. M. Massie, E. Mächler, G. M. Palamara, M. Seymour, O. L. Petchey, Biodiversity increases and decreases ecosystem stability. *Nature* **563**, 109–112 (2018).

64. D. Tilman, J. A. Downing, Biodiversity and stability in grasslands. *Nature* **367**, 363–365 (1994).

65. J. Van Ruijven, F. Berendse, Diversity enhances community recovery, but not resistance, after drought. *J. Ecol.* **98**, 81–86 (2010).

66. P. Vasilakopoulos, C. T. Marshall, Resilience and tipping points of an exploited fish population over six decades. *Glob. Chang. Biol.* **21**, 1834–1847 (2015).

67. V. Radchuk, F. D. Laender, J. S. Cabral, I. Boulangeat, M. Crawford, F. Bohn, J. D. Raedt, C. Scherer, J. Svenning, K. Thonicke, F. M. Schurr, V. Grimm, S. Kramer-Schadt, The dimensionality of stability depends on disturbance type. *Ecol. Lett.* **22**, 674–684 (2019).

68. J. J. Heymans, M. Coll, S. Libralato, L. Morissette, V. Christensen, Global patterns in ecological indicators of marine food webs: A modelling approach. *PLOS ONE* **9**, e95845 (2014).

69. I. Keramidas, D. Dimarchopoulou, A. C. Tsikliras, Modelling and assessing the ecosystem of the Aegean Sea, a major hub of the eastern Mediterranean at the intersection of Europe and Asia. *Reg. Stud. Mar. Sci.* **56**, 102704 (2022).

70. A. J. T. Dalsgaard, D. Pauly, T. A. Okey, Preliminary mass-balance model of Prince William Sound, Alaska, for the pre-spill period, 1980–1989 (1997). https://doi.org/10.14288/1.0074775.

71. T. Okey, *A Trophodynamic Model of Albatross Bay*, *Gulf of Carpentaria: Revealing a Plausible Fishing Explanation for Prawn Catch Declines* (2006).

72. S. Guénette, S. J. Heymans, V. Christensen, A. W. Trites, Ecosystem models show combined effects of fishing, predation, competition, and ocean productivity on Steller sea lions (*Eumetopias jubatus*) in Alaska. *Can. J. Fish. Aquat. Sci.* **63**, 2495–2517 (2006).

73. M. V. Morales-Zárate, F. Arreguín-Sánchez, J. López-Martínez, S. E. Lluch-Cota, Ecosystem trophic structure and energy flux in the Northern Gulf of California, México. *Ecol. Model.* **174**, 331–345 (2004).

74. K. Allen, J. A. Garwood, K. Hu, E. A. Meselhe, K. A. Lewis, Simulating synergistic impacts of climate change and human induced stressors on a northern Gulf of Mexico estuarine food web. *Front. Mar. Sci.* **10**, 10.3389/fmars.2023.1213949 (2023).

75. D. Lercari, L. Bergamino, O. Defeo, Trophic models in sandy beaches with contrasting morphodynamics: Comparing ecosystem structure and biomass flow. *Ecol. Model.* **221**, 2751–2759 (2010).

76. T. Morato, E. Lemey, G. Menezes, C. K. Pham, J. Brito, A. Soszynski, T. J. Pitcher, J. J. Heymans, Food-web and ecosystem structure of the open-ocean and deep-sea environments of the Azores, NE Atlantic. *Front. Mar. Sci.* **3**, 10.3389/fmars.2016.00245 (2016).

77. S. Guénette, V. Christensen, D. Pauly, Fisheries impacts on North Atlantic ecosystems: Models and analyses (2001); https://doi.org/10.14288/1.0348145.

78. G. Halouani, C.-M. Villanueva, A. Raoux, J. C. Dauvin, F. Ben Rais Lasram, E. Foucher, F. Le Loc'h, G. Safi, E. Araignous, J. P. Robin, N. Niquil, A spatial food web model to investigate potential spillover effects of a fishery closure in an offshore wind farm. *J. Mar. Syst.* **212**, 103434 (2020).

79. A. M. Cisneros-Montemayor, V. Christensen, F. Arreguín-Sánchez, U. R. Sumaila, Ecosystem models for management advice: An analysis of recreational and commercial fisheries policies in Baja California Sur, Mexico. *Ecol. Model.* **228**, 8–16 (2012).

80. M. Colléter, D. Gascuel, J.-M. Ecoutin, L. Tito De Morais, Modelling trophic flows in ecosystems to assess the efficiency of marine protected area (MPA), a case study on the coast of Sénégal. *Ecol. Model.* **232**, 1–13 (2012).

81. J. Blanchard, J. Pinnegar, S. Mackinson, Exploring marine mammal-fishery interactions using "Ecopath with Ecosim": Modelling the Barents Sea ecosystem. *Sci. Ser. Tech. Rep.* **117**, (2002).

82. J. M. Vasslides, H. Townsend, T. Belton, O. P. Jensen, Modeling the effects of a power plant decommissioning on an estuarine food web. *Estuaries Coasts* **40**, 604–616 (2017).

83. F. Moullec, D. Gascuel, K. Bentorcha, S. Guénette, M. Robert, Trophic models: What do we learn about celtic sea and bay of biscay ecosystems? *J. Mar. Syst.* **172**, 104–117 (2017).

84. C. H. Ainsworth, J. J. Heymans, T. Pitcher, M. Vasconcellos, Ecosystem models of Northern British Columbia for the time periods 2000, 1950, 1900 and 1750 (2002). https://doi.org/10.14288/1.0074796.

85. G. Lassalle, D. Gascuel, F. Le Loc'h, J. Lobry, G. J. Pierce, V. Ridoux, M. B. Santos, J. Spitz, N. Niquil, An ecosystem approach for the assessment of fisheries impacts on marine top predators: The Bay of Biscay case study: the Bay of Biscay case study. *ICES J. Mar. Sci.* **69**, 925–938 (2012).

86. A. C. Gucu, Can overfishing be responsible for the successful establishment of *Mnemiopsis leidyi* in the Black Sea? *Estuar. Coast. Shelf Sci.* **54**, 439–451 (2002).

87. E. Akoglu, B. Salihoglu, S. Libralato, T. Oguz, C. Solidoro, An indicator-based evaluation of Black Sea food web dynamics during 1960–2000. *J. Mar. Syst.* **134**, 113–125 (2014).

88. D. B. Preikshot, "The influence of geographic scale, climate and trophic dynamics upon North Pacific oceanic ecosystem models," thesis, University of British Columbia (2007).

89. K. John, N. V. C. Pinnegar, Polunin, predicting indirect effects of fishing in mediterranean rocky littoral communities using a dynamic simulation model. *Ecol. Model.* **172**, 249–267 (2004).

90. T. J. Pitcher, M. Vasconcellos, J. J. Heymans, C. Brignall, N. Haggan, Information supporting past and present ecosystem models of Northern British Columbia and the Newfoundland shelf (2002). https://doi.org/10.14288/1.0348150.

91. X. Corrales, D. Vilas, C. Piroddi, J. Steenbeek, J. Claudet, J. Lloret, A. Calò, A. Di Franco, T. Font, A. Ligas, G. Prato, R. Sahyoun, P. Sartor, P. Guidetti, M. Coll, Multi-zone marine protected areas: Assessment of ecosystem and fisheries benefits using multiple ecosystem models. *Ocean Coast. Manag.* **193**, 105232 (2020).

92. L. Morissette, J. L. Melgo, K. Kaschner, L. Gerber, Modelling the trophic role of marine mammals in tropical areas: Data requirements, uncertainty, and validation (2009). https://doi.org/10.14288/1.0074740.

93. M. E. Vega-Cendejas, F. Arreguín-Sánchez, Energy fluxes in a mangrove ecosystem from a coastal lagoon in Yucatan Peninsula, Mexico. *Ecol. Model.* **137**, 119–133 (2001).

94. P.-Y. Hernvann, D. Gascuel, A. Grüss, J.-N. Druon, D. Kopp, I. Perez, C. Piroddi, M. Robert, The Celtic sea through time and space: Ecosystem modeling to unravel fishing and climate change impacts on food-web structure and dynamics. *Front. Mar. Sci.* **7**, 578717 (2020).

95. A. Bentorcha, D. Gascuel, S. Guénette, Using trophic models to assess the impact of fishing in the Bay of Biscay and the Celtic Sea. *Aquat. Living Resour.* **30**, 7 (2017).

96. M. L. D. Palomares, D. Pauly, Philippine marine fisheries catches: A bottom-up reconstruction, 1950 to 2010 (2014). https://doi.org/10.14288/1.0354317.

97. M. T. Tomczak, S. Niiranen, O. Hjerne, T. Blenckner, Ecosystem flow dynamics in the Baltic Proper—Using a multi-trophic dataset as a basis for food–web modelling. *Ecol. Model.* **230**, 123–147 (2012).

98. S. Neira, H. Arancibia, L. Cubillos, Comparative analysis of trophic structure of commercial fishery species off Central Chile in 1992 and 1998. *Ecol. Model.* **172**, 233–248 (2004).

99. F. Arreguín-Sánchez, E. Arcos, E. A. Chávez, Flows of biomass and structure in an exploited benthic ecosystem in the gulf of California, Mexico. *Ecol. Model.* **156**, 167–183 (2002).

100. J. M. López-Vila, J. J. Schmitter-Soto, E. Velázquez-Velázquez, E. Barba-Macías, I. H. Salgado-Ugarte, Young does not mean unstable: A trophic model for an estuarine lagoon system in the Southern Mexican pacific. *Hydrobiologia* **827**, 225–246 (2019).

101. V. Christensen, Fisheries ecosystem model of the Chesapeake Bay methodology, parameterization, and model exploration (2009).

102. M. P. Dias, R. Martin, E. J. Pearmain, I. J. Burfield, C. Small, R. A. Phillips, O. Yates, B. Lascelles, P. G. Borboroglu, J. P. Croxall, Threats to seabirds: A global assessment. *Biol. Conserv.* **237**, 525–537 (2019).

103. N. Michailidis, X. Corrales, P. K. Karachle, N. Chartosia, S. Katsanevakis, S. Sfenthourakis, Modelling the role of alien species and fisheries in an Eastern Mediterranean insular shelf ecosystem. *Ocean Coast. Manag.* **175**, 152–171 (2019).

104. R. T. M. Bacalso, M. Wolff, Trophic flow structure of the Danajon ecosystem (Central Philippines) and impacts of illegal and destructive fishing practices. *J. Mar. Syst.* **139**, 103–118 (2014).

105. S. Tecchio, M. Coll, V. Christensen, J. B. Company, E. Ramírez-Llodra, F. Sardà, Food web structure and vulnerability of a deep-sea ecosystem in the NW Mediterranean Sea. *Deep Sea Res.* **75**, 1–15 (2013).

106. D. Zeller, J. Reinert, Modelling spatial closures and fishing effort restrictions in the Faroe Islands marine ecosystem. *Ecol. Model.* **172**, 403–420 (2004).

107. A. W. Trites, P. A. Livingston, S. Mackinson, M. Vasconcellos, A. M. Springer, D. (Daniel) Pauly, Ecosystem change and the decline of marine mammals in the eastern bering sea: Testing the ecosystem shift and commercial whaling hypotheses (1999). https://doi.org/10.14288/1.0348097.

108. L. Vanalderweireldt, C. Albouy, F. Le Loc'h, R. Millot, C. Blestel, M. Patrissi, M. Marengo, J. Garcia, C. Bousquet, C. Barrier, M. Lefur, P.-A. Bisgambiglia, A. Donnay, S. Ternengo, A. Aiello, P. Lejeune, E. D. H. Durieux, Ecosystem modelling of the Eastern Corsican Coast (ECC): Case study of one of the least trawled shelves of the Mediterranean Sea. *J. Mar. Syst.* **235**, 103798 (2022).

109. R. J. Olson, G. M. Watters, A model of the pelagic ecossystem in the eastern tropical Pacific Ocean. *Inter. Am. Trop. Tuna Comm. Bull.* **22**, 135–218 (2021).

110. I. Tsehaye, L. A. J. Nagelkerke, Exploring optimal fishing scenarios for the multispecies artisanal fisheries of Eritrea using a trophic model. *Ecol. Model.* **212**, 319–333 (2008).

111. M. Smith, D. Chagaris, R. Paperno, S. Markwith, Ecosystem structure and resilience of the Florida Bay Estuary: An original ecosystem model with implications for everglades restoration. *Mar. Freshw. Res.* **72**, 563 (2021).

112. D. J. Ruiz, S. Banks, M. Wolff, Elucidating fishing effects in a large-predator dominated system: The case of Darwin and Wolf Islands (Galápagos). *J. Sea Res.* **107**, 1–11 (2016).

113. T. Okey, A trophic model of a Galápagos subtidal rocky reef for evaluating fisheries and conservation strategies. *Ecol. Model.* **172**, 383–401 (2004).

114. J. S. Link, C. A. Griswold, E. T. Methratta, J. Gunnard, J. K. T. Brodziak, L. A. Col, D. D. Dow, S. F. Edwards, M. J. Fogarty, S. A. Fromm, J. R. Green, V. G. Guida, D. Johnson, J. M. Kane, C. M. Legault, J. E. O'Reilly, W. J. Overholtz, D. L. Palka, W. T. Stockhausen, J.

J. Vitaliano, G. T. Waring, Documentation for the Energy Modeling and Analysis eXercise (EMAX). (2006); https://repository.library.noaa.gov/view/noaa/5277.

115. M. Wolff, H. J. Hartmann, V. Koch, A pilot trophic model for Golfo Dulce, a fjord-like tropical embayment, Costa Rica. *Rev. Biol. Trop.* **44**, 215–231 (1996).

116. J. J. Heymans, Ecosystem models of Newfoundland and Southeastern Labrador: Additional information and analyses for "back to the future" (2003). https://doi.org/10.14288/1.0074790.

117. D. Gascuel, S. Guénette, I. Diallo, A. Sidibé, Impact de la pêche sur l'écosystème marin de Guinée - modélisation EwE 1985/2005 (2009). [Fishing impact on the marine Guinean ecosystem: a 1985/2005 model using EwE]. https://doi.org/10.14288/1.0074751.

118. D. Lercari, F. Arreguín-Sánchez, An ecosystem modelling approach to deriving viable harvest strategies for multispecies management of the Northern Gulf of California. *Aquat. Conserv.* **19**, 384–397 (2009).

119. T. Hattab, F. Ben Rais Lasram, C. Albouy, M. S. Romdhane, O. Jarboui, G. Halouani, P. Cury, F. Le Loc'h, An ecosystem model of an exploited southern Mediterranean shelf region (Gulf of Gabes, Tunisia) and a comparison with other Mediterranean ecosystem model properties. *J. Mar. Syst.* **128**, 159–174 (2013).

120. C. Walters, S. Martell, B. Mahmoudi, An Ecosim model for exploring ecosystem management options for the Gulf of Mexico: Implications of including multistanza life history models for policy Predictions. *Bull. Mar. Sci.* **83**, 251–271 (2006).

121. M. Wolff, V. Koch, J. Chavarría, J. Vargas, A trophic flow model of the Golfo de Nicoya, Costa Rica. *Rev. de Biol Trop.* **46**, 63–79 (1998).

122. L. O. Duarte, C. B. García, Trophic role of small pelagic fishes in a tropical upwelling ecosystem. *Ecol. Model.* **172**, 323–338 (2004).

123. V. Christensen, Fishery-induced changes in a marine ecosystem: Insight from models of the Gulf of Thailand. *J. Fish Biol.* **53**, 128–142 (1998).

124. C. Hoover, "Hudson bay ecosystem: Past, present, and future" in *A Little Less Arctic*, S. H. Ferguson, L. L. Loseto, M. L. Mallory, Eds. (Springer Netherlands, 2010), pp. 217–236.

125. M. J. Zetina-Rejón, F. Arreguín-Sánchez, E. A. Chávez, Trophic structure and flows of energy in the Huizache–Caimanero lagoon complex on the Pacific coast of Mexico. *Estuar. Coast. Shelf Sci.* **57**, 803–815 (2003).

126. J. Tam, M. H. Taylor, V. Blaskovic, P. Espinoza, R. Michael Ballón, E. Díaz, C. Wosnitza-Mendo, J. Argüelles, S. Purca, P. Ayón, L. Quipuzcoa, D. Gutiérrez, E. Goya, N. Ochoa, M. Wolff, Trophic modeling of the Northern Humboldt current ecosystem, Part I: Comparing trophic linkages under La Niña and El Niño conditions. *Prog. Oceanogr.* **79**, 352–365 (2008).

127. M. H. Taylor, M. Wolff, J. Mendo, C. Yamashiro, Changes in trophic flow structure of Independence Bay (Peru) over an ENSO cycle. *Prog. Oceanogr.* **79**, 336–351 (2008).

128. K. Lees, S. Mackinson, An Ecopath model of the Irish Sea: Ecosystems properties and sensitivity. (2007).

129. H. M. Lozano-Montes, N. R. Loneragan, R. C. Babcock, K. Jackson, Using trophic flows and ecosystem structure to model the effects of fishing in the Jurien Bay Marine Park, temperate Western Australia. *Mar. Freshw. Res.* **62**, 421 (2011).

130. C. Wabnitz, G. Balazs, S. Beavers, K. Bjorndal, A. Bolten, V. Christensen, S. Hargrove, D. Pauly, Ecosystem structure and processes at Kaloko Honokohau, focusing on the role of herbivores, including the green sea turtle Chelonia mydas, in reef resilience. *Mar. Ecol. Prog. Ser.* **420**, 27–44 (2010).

131. H.-J. Lin, K.-T. Shao, J.-S. Hwang, W.-T. Lo, I.-J. Cheng, L.-H. Lee, A trophic model for Kuosheng Bay in Northern Taiwan. *J. Mar. Sci. Technol.* **12**, (2004).

132. H.-J. Lin, K.-T. Shao, S.-R. Kuo, H.-L. Hsieh, S.-L. Wong, I.-M. Chen, W.-T. Lo, J.-J. Hung, A trophic model of a sandy Barrier Lagoon at Chiku in Southwestern Taiwan. *Estuar. Coast. Shelf Sci.* **48**, 575–588 (1999).

133. J. M. Venier, “Seasonal ecosystem models of the Looe Key National Marine Sanctuary, Florida,” thesis, University of British Columbia (1997).

134. D. Vilas, M. Coll, T. Pedersen, X. Corrales, K. Filbee-Dexter, M. F. Pedersen, K. M. Norderhaug, S. Fredriksen, T. Wernberg, E. Ramírez-Llodra, Kelp-carbon uptake by Arctic deep-sea food webs plays a noticeable role in maintaining ecosystem structural and functional traits. *J. Mar. Syst.* **203**, 103268 (2020).

135.. Saygu, E. Akoglu, G. Gül, D. Bedikoğlu, N. Demirel, Fisheries impact on the Sea of Marmara ecosystem structure and functioning during the last three decades. *Front. Mar. Sci.* **9**, 1076399 (2023).

136. S. Guénette, B. Meissa, D. Gascuel, Assessing the contribution of marine protected areas to the trophic functioning of ecosystems: A model for the Banc d’Arguin and the mauritanian shelf. *PLOS ONE* **9**, e94742 (2014).

137. S. Libralato, M. Tempesta, C. Solidoro, M. Spoto, An ecosystem model applied to Miramare Natural Marine Reserve: Limits, advantages and perspectives. *Biol. Mar. Mediterr.* **13**, 386–395 (2006).

138. E. N. Fondo, M. Chaloupka, J. J. Heymans, G. A. Skilleter, Banning fisheries discards abruptly has a negative impact on the population dynamics of charismatic marine Megafauna. *PLOS ONE* **10**, e0144543 (2015).

139. F. Arbach Leloup, N. Desroy, P. Le Mao, D. Pauly, O. Le Pape, Interactions between a natural food web, shellfish farming and exotic species: The case of the Bay of Mont Saint Michel (France). *Estuar. Coast. Shelf Sci.* **76**, 111–120 (2008).

140. A. Innes-Gold, M. Heinichen, K. Gorospe, C. Truesdale, J. Collie, A. Humphries, Modeling 25 years of food web changes in Narragansett Bay (USA) as a tool for ecosystem-based management. *Mar. Ecol. Prog. Ser.* **654**, 17–33 (2020).

141. C. H. Ainsworth, U. R. Sumaila, Intergenerational valuation of fisheries resources can justify long-term conservation: A case study in Atlantic cod (*Gadus morhua*). *Can. J. Fish. Aquat. Sci.* **62**, 1104–1110 (2005).

142. K. Tsagarakis, M. Coll, M. Giannoulaki, S. Somarakis, C. Papaconstantinou, A. Machias, Food-web traits of the North Aegean Sea ecosystem (Eastern Mediterranean) and comparison with other Mediterranean ecosystems. *Estuar. Coast. Shelf Sci.* **88**, 233–248 (2010).

143. K. Watermeyer, L. Shannon, J.-P. Roux, C. Griffiths, Changes in the trophic structure of the northern Benguela before and after the onset of industrial fishing. *Afr. J. Mar. Sci.* **30**, 383–403 (2008).

144. M. Wolff, V. Koch, V. Isaac, A trophic flow model of the Caeté Mangrove Estuary (North Brazil) with considerations for the sustainable use of its resources. *Estuar. Coast. Shelf Sci.* **50**, 789–803 (2000).

145. J. Field, Application of ecosystem-based fishery management approaches in the Northern California Current (2004).

146. S. R. Sagarese, M. V. Lauretta, J. F. Walter, Progress towards a next-generation fisheries ecosystem model for the northern Gulf of Mexico. *Ecol. Model.* **345**, 75–98 (2017).

147. C. Savenkoff, H. Bourdages, M. Castonguay, L. Morissette, D. Chabot, M. Hammill, Input data and parameter estimates for ecosystem models of the northern Gulf of St. Lawrence (mid-1990s) (2004).

148. L. Morissette, S.-P. Despatie, C. Savenkoff, Data gathering and input parameters to construct ecosystem models for the northern Gulf of St. Lawrence (mid-1980s). *Can. Tech. Rep. Fish. Aquat. Sci.* **2497**, (2003).

149. S. Mackinson, G. Daskalov, An ecosystem model of the North Sea to support an ecosystem approach to fisheries management: Description and parameterisation. *Sci. Ser Tech. Rep.* **142**, (2007).

150. W. L. Cheung, “Vulnerability of marine fishes to fishing: From global overview to the northern South China Sea,” thesis, University of British Columbia (2007).

151. L. Morissette, K. Kaschner, L. R. Gerber, ‘Whales eat fish’? Demystifying the myth in the Caribbean marine ecosystem. *Fish Fish.* **11**, 388–404 (2010).

152. V. Brando, Assessment of environmental management effects in a shallow water basin using mass-balance models. *Ecol. Model.* **172**, 213–232 (2004).

153. D. Dimarchopoulou, I. Keramidas, K. Tsagarakis, A. C. Tsikliras, Ecosystem models and effort simulations of an untrawled gulf in the central aegean sea. *Front. Mar. Sci.* **6**, 648 (2019).

154. R. Angelini, A. A. Agostinho, Food web model of the Upper Paraná River floodplain: Description and aggregation effects. *Ecol. Model.* **181**, 109–121 (2005).

155. S. Guénette, V. Christensen, D. Pauly, Trophic modelling of the Peruvian upwelling ecosystem: Towards reconciliation of multiple datasets. *Prog. Oceanogr.* **79**, 326–335 (2008).

156. T. Pedersen, M. Fuhrmann, U. Lindstrøm, E. Nilssen, T. Ivarjord, V. Ramasco, L. Jørgensen, J. Sundet, K. Sivertsen, E. Källgren, A. Hjelset, C. Michaelsen, G. Systad, F. Norrbin, M. Svenning, A. Bjørge, H. Steen, K. Nilssen, Effects of the invasive red king crab on food web structure and ecosystem properties in an Atlantic fjord. *Mar. Ecol. Prog. Ser.* **596**, 13–31 (2018).

157. A. Valls, D. Gascuel, S. Guénette, P. Francour, Modeling trophic interactions to assess the effects of a marine protected area: Case study in the NW Mediterranean Sea. *Mar. Ecol. Prog. Ser.* **456**, 201–214 (2012).

158. G. Prato, C. Barrier, P. Francour, V. Cappanera, V. Markantonatou, P. Guidetti, L. Mangialajo, R. Cattaneo-Vietti, D. Gascuel, Assessing interacting impacts of artisanal and recreational fisheries in a small Marine Protected Area (Portofino, NW Mediterranean Sea). *Ecosphere* **7**, e01601 (2016).

159. T. J. Pitcher, K. L. Cochrane, The use of ecosystem models to investigate multispecies management strategies for capture fisheries (2002). https://doi.org/10.14288/1.0348151.

160. T. Okey, B. Wright, Toward ecosystem-based extraction policies for Prince William Sound, Alaska: Integrating conflicting objectives and rebuilding pinnipeds. *Bull. Mar. Sci.* **74**, 727–747 (2004).

161. T. J. Pitcher, C. H. Ainsworth, M. Bailey, Ecological and economic analyses of marine ecosystems in the Bird's Head Seascape, Papua, Indonesia: I (2007). https://doi.org/10.14288/1.0074756.

162. S. Gamito, K. Erzini, Trophic food web and ecosystem attributes of a water reservoir of the Ria Formosa (south Portugal). *Ecol. Model.* **181**, 509–520 (2005).

163. L. Capitani, R. Angelini, F. W. Keppeler, G. Hallwass, R. A. M. Silvano, Food web modeling indicates the potential impacts of increasing deforestation and fishing pressure in the Tapajós River, Brazilian Amazon. *Reg. Environ. Change* **21**, 42 (2021).

164. J. T. Bayle-Sempere, F. Arreguín-Sánchez, P. Sanchez-Jerez, L. A. Salcido-Guevara, D. Fernandez-Jover, M. J. Zetina-Rejón, Trophic structure and energy fluxes around a Mediterranean fish farm. *Ecol. Model.* **248**, 135–147 (2013).

165. E. Rivera, A. Lara, G. Villalobos, A. Yáñez-Arancibia, Trophodynamic ecology of two critical habitats (seagrasses and mangroves) in Términos Lagoon, southern Gulf of Mexico. *Fish Cent. Res. Rep.* **11**, 245–254 (2003).

166. M. H. Taylor, M. Wolff, F. Vadas, C. Yamashiro, Trophic and environmental drivers of the Sechura Bay Ecosystem (Peru) over an ENSO cycle. *Helgol. Mar. Res.* **62**, 15–32 (2008).

167. M. L. D. Palomares, D. (Daniel) Pauly, West African marine ecosystems: Models and fisheries impacts. (2004). https://doi.org/10.14288/1.0074805.

168. A. Lira, R. Angelini, F. Le Loc'h, F. Ménard, C. Lacerda, T. Frédou, F. L. Frédou, Trophic flow structure of a neotropical estuary in northeastern Brazil and the comparison of ecosystem model indicators of estuaries. *J. Mar. Syst.* **182**, 31–45 (2018).

169. L. A. V. Bezerra, R. Angelini, J. R. S. Vitule, M. Coll, J. I. Sánchez-Botero, Food web changes associated with drought and invasive species in a tropical semiarid reservoir. *Hydrobiologia* **817**, 475–489 (2018).

170. M. Zetina-Rejón, F. Arreguín-Sánchez, "Flujos de energía y estructura trófica de la Sonda de Campeche, Suroeste del Golfo de México" in *Memorias Del III Foro de Camarón Del Golfo de México y Del Mar Caribe,* (INP-SAGARPA y Gob. del Estadode Campeche, México, 2003), pp. 55–62. [Energy flows and trophic structure of the Campeche Sound, southwestern Gulf of Mexico].

171. S. Manickchand-Heileman, L. A. Soto, E. Escobar, A preliminary trophic model of the continental shelf, South-western gulf of Mexico. *Estuar. Coast. Shelf Sci.* **46**, 885–899 (1998).

172. J. Falk-Petersen, "Ecosystem effects of red king crab invasion. A modelling approach using Ecopath with Ecosim," thesis, Universitetet i Tromsø (2004).

173. L. J. Shannon, C. L. Moloney, A. Jarre, J. G. Field, Trophic flows in the southern Benguela during the 1980s and 1990s. *J. Mar. Syst.* **39**, 83–116 (2003).

174. M. Vasconcellos, M. A. Gasalla, Fisheries catches and the carrying capacity of marine ecosystems in southern Brazil. *Fish. Res.* **50**, 279–295 (2001).

175. E. L. Bredesen, "Krill and the Antarctic: Finding the balance," thesis, University of British Columbia (2003).

176. F. Arreguín-Sánchez, E. Valero-Pacheco, E. Chávez, "A trophic box model of the coastal fish communities of the southwestern Gulf of Mexico" in *Trophic models of aquatic ecosystems*. *ICLARM Conf. Proc.* **26**, 197–205 (1993).

177. V. Christensen, L. R. Garces, G. Silvestre, D. Pauly, *Fisheries Impact on the South China Sea Large Marine Ecosystem: A Preliminary Analysis Using Spatially-Explicit Methodology* (WorldFish, 2003).

178. S. S. K. Haputhantri, M. C. S. Villanueva, J. Moreau, Trophic interactions in the coastal ecosystem of Sri Lanka: An ECOPATH preliminary approach. *Estuar. Coast. Shelf Sci.* **76**, 304–318 (2008).

179. S. Martell, A. Beattie, C. Walters, "Simulating fisheries management strategies in the Strait of Georgia ecosystem using Ecopath and Ecosim" in *The Use of Ecosystem Models to Investigate Multispecies Management Strategies for Capture Fisheries* (Fisheries Centre, University of British Columbia, 2002), vol. 10, pp. 16–24.

180. R. A. Watson, G. B. Nowara, S. R. Tracey, E. A. Fulton, C. M. Bulman, G. J. Edgar, N. S. Barrett, J. M. Lyle, S. D. Frusher, C. D. Buxton, Ecosystem model of Tasmanian waters explores impacts of climate-change induced changes in primary productivity. *Ecol. Model.* **264**, 115–129 (2013).

181. S. Manickchand-Heileman, F. Arreguín-Sánchez, A. Lara-Domínguez, L. A. Soto, Energy flow and network analysis of Terminos Lagoon, SW Gulf of Mexico. *J. Fish Biol.* **53**, 179–197 (1998).

182. D. Dimarchopoulou, K. Tsagarakis, G. Sylaios, A. C. Tsikliras, Ecosystem trophic structure and fishing effort simulations of a major fishing ground in the northeastern Mediterranean Sea (Thermaikos Gulf). *Estuar. Coast. Shelf Sci.* **264**, 107667 (2022).

183. S. Opitz, *Trophic Interactions in Caribbean Coral Reefs* (International Center for Living Aquatic Resources Management, 1996), *ICLARM contribution*.

184. S. Pedro, M. Lemire, C. Hoover, B. Saint-Béat, M. Y. Janjua, J. Herbig, M. Geoffroy, G. Yunda-Guarin, M.-A. Moisan, J. Boissinot, J.-É. Tremblay, M. Little, L. Chan, M. Babin, T.-A. Kenny, F. Maps, Structure and function of the western Baffin Bay coastal and shelf ecosystem. *Elementa Sci. Anthrop.* **11**, 00015 (2023).

185. G. Len, A. Man, A. Talib, M. Mohamad-Norizam, G. T. Silvestre, "A Trophic Model of the Coastal Fisheries Ecosystem off the West Coast of Sabah and Sarawak, Malaysia" in *Assessment, Management and Future Directions for Coastal Fisheries in Asian Countries*. *WorldFish Center Conf. Proc.* **67**, 1120 (2023).

186. S. J. D. Martell, “Variation in pink shrimp populations off the west coast of Vancouver Island: Oceanographic and trophic interactions,” thesis, University of British Columbia (2002).

187. A. Dahood, K. De Mutsert, G. M. Watters, Evaluating Antarctic marine protected area scenarios using a dynamic food web model. *Biol. Conserv.* **251**, 108766 (2020).

188. K. Y. Aydin, V. V. Lapko, V. I. Radchenko, P. A. Livingston, A Comparison of the eastern Bering and western Bering Sea shelf and slope ecosystems through the use of mass-balance food web models (2002).

189. O. Godinot, V. Allain, A preliminary Ecopath model of the warm pool pelagic ecosystem, in *16th Meeting of the Standing Committee on Tuna and Billfish* (2003).

190. D. D. Chagaris, B. Mahmoudi, C. J. Walters, M. S. Allen, Simulating the trophic impacts of fishery policy options on the West Florida shelf using ecopath with ecosim. *Mar. Coast. Fish* **7**, 44–58 (2015).

191. N. Haggan, T. Pitcher, *Ecosystem simulation models of Scotland's West Coast and sea lochs* (2005). https://doi.org/10.14288/1.0074795.

192. F. Arreguín-Sánchez, J. C. Seijo, E. Valero-Pacheco, An application of ECOPATH II to the north continental shelf ecosystem of Yucatan, Mexico. *ICLARM Conf. Proc.* **26**, 269–278 (1993).
